# Butyrophilin-like proteins display combinatorial diversity in selecting and maintaining signature intraepithelial γδ T cell compartments

**DOI:** 10.1038/s41467-020-17557-y

**Published:** 2020-07-28

**Authors:** Anett Jandke, Daisy Melandri, Leticia Monin, Dmitry S. Ushakov, Adam G. Laing, Pierre Vantourout, Philip East, Takeshi Nitta, Tomoya Narita, Hiroshi Takayanagi, Regina Feederle, Adrian Hayday

**Affiliations:** 1 Immunosurveillance Laboratory, The Francis Crick Institute, 1 Midland Road, London, NW11AT UK; 2 Peter Gorer Department of Immunobiology, School of Immunology and Microbial Sciences, King’s College London, Great Maze Pond, London Bridge, London, SE19RT UK; 3 Bioinformatics and Biostatistics Laboratory, The Francis Crick Institute, 1 Midland Road, London, NW11AT UK; 4 Department of Immunology, Graduate School of Medicine and Faculty of Medicine, University of Tokyo, Hongo 7-3-1, Bunkyo-ku, Tokyo 113-0033 Japan; 5 Department of Pharmacotherapy, Research Institute of Pharmaceutical Sciences, Musashino University, Nishitokyo, Tokyo 202-8585 Japan; 6 Monoclonal Antibody Core Facility, Institute for Diabetes and Obesity, Helmholtz Zentrum, München, German Research Centre for Environmental Health, 85764 Neuherberg, Germany

**Keywords:** Immunology, Cellular immunity, Gammadelta T cells, Mucosal immunology

## Abstract

Butyrophilin-like (*Btnl*) genes are emerging as major epithelial determinants of tissue-associated γδ T cell compartments. Thus, the development of signature, murine TCRγδ^+^ intraepithelial lymphocytes (IEL) in gut and skin depends on *Btnl* family members, *Btnl1* and *Skint1*, respectively. In seeking mechanisms underlying these profound effects, we now show that normal gut and skin γδ IEL development additionally requires *Btnl6* and *Skint2*, respectively, and furthermore that different Btnl heteromers can seemingly shape different intestinal γδ^+^ IEL repertoires. This formal genetic evidence for the importance of Btnl heteromers also applied to the steady-state, since sustained Btnl expression is required to maintain the signature TCR.Vγ7^+^ IEL phenotype, including specific responsiveness to Btnl proteins. In sum, Btnl proteins are required to select and to maintain the phenotypes of tissue-protective γδ IEL compartments, with combinatorially diverse heteromers having differential impacts on different IEL subsets.

## Abstract (web summary)

Butyrophilin-like genes are emerging as central to tissue associated γδ T cell compartments. Here, the authors show that the butyropilin-like gene-products exert their effects as combinatorially diverse heteromers that differentially affect the selection and maintenance of skin-resident and gut-resident intraepithelial γδ T-cell populations.

## Introduction

From jawless vertebrates through to humans, many extralymphoid tissues harbour distinct immune cell populations. Those include tissue-resident memory (*T*_RM_) cells that infiltrate tissues after antigen priming in lymphoid tissues, and remain well-placed to respond to local antigen recurrence^1^. In addition, various myeloid and lymphoid cells, including macrophages^2^, T-regulatory cells^3^ and γδ T cells become associated with tissues developmentally, remaining in situ lifelong^4–8^. Such cells are implicated in protecting tissue integrity, and γδ T-cell deficiency is causally linked to cancer, tissue inflammation and defective wound healing^9–12^.

In addition, the molecular phenotypes of local T cells commonly emphasise their relationships to specific anatomical sites^3^. Thus, murine γδ T cells, which were revealed some 30 years ago to be prototypic tissue-associated T cells, display tissue-restricted T-cell receptor (TCR) repertoires, including Vγ5Vδ1 in the epidermis, Vγ6Vδ1 in the uterus, dermis and lung, and Vγ7^+^ cells expressing a variety of Vδ chains in the small intestine^13^. Nonetheless, how such TCRs contributed to tissue protection remained enigmatic, particularly given that Vγ5Vδ1^+^ dendritic epidermal T cells (DETC) were shown to use innate receptors, specifically NKG2D, to respond rapidly to epithelial cell dysregulation^14,15^.

Recently however, signature TCRs were shown to mediate the tissue-specific selection of γδ T cells by members of the heretofore enigmatic butyrophilin-like (Btnl) subfamily of B7 genes. Thus, *Btnl1*^*−/−*^ mice mostly lack intestinal Vγ7^+^ cells^16^, while mice deficient in *Skint1* (a *Btnl*-related gene) specifically lack Vγ5Vδ1^+^ DETC^17,18^. The conservation of this biology became evident when human colonic Vγ4^+^ cells were shown to be specifically regulated by BTNL3^16,19^, while Butyrophilin 3A1 (BTN3A1) and BTN2A1^20^ were found to be critical for signature responses of human peripheral blood Vγ9Vδ2^+^ cells to low molecular mass phosphoantigens such as isopentenyl pyrophosphate^21–23^. Moreover, dysregulation of the BTNL3-Vγ4^+^ axis has been implicated in celiac disease^24^. Thus, there is considerable interest in the mechanisms by which *Skint/Btnl*/*BTN* genes exert their effects.

Consistent with their regulation of γδ T-cell subsets defined by their TCRs, Btnl/BTNL proteins have emerged as *bona fide* T-cell selecting ligands akin to MHC or CD1. In addition, evidence from cell culture and biochemical experiments argues that Btnl/BTNL/BTN proteins may exert their impacts as heterodimers of Btnl1 + 6, BTNL3 + 8, and BTN3A1 + 2A1, respectively^16,19,20,25^. Nonetheless, the functional significance of heteromers has not been universally accepted^26^, with one concern being that the most compelling evidence is based on cellular over-expression systems^27,28^.

This study has addressed this important issue by use of genetics. By showing that Vγ5Vδ1^+^ DETC development depends on *Skint2* as well as on *Skint1*, and that Vγ7^+^ intestinal IEL development depends on *Btnl6* as well as on *Btnl1*, we now provide formal genetic evidence that single Btnls are not sufficient for IEL selection. Most unexpectedly, however, different Btnl pairings had differential effects on IEL with different TCRs, revealing a potential for combinatorial diversity that could finely tune IEL repertoire composition. The major impacts of *Skint1* and *Btnl1* on IEL maturation occur during narrow time-windows in early life. Beyond this, the sustained expression of *Btnl* genes is herewith shown to be required to maintain signature intestinal IEL phenotypes. In sum, epithelial Btnl proteins mediate a sustained and complex regulation of local γδ T-cell compartments.

## Results

### DETC development requires *Skint2*

**Fig. 1:**
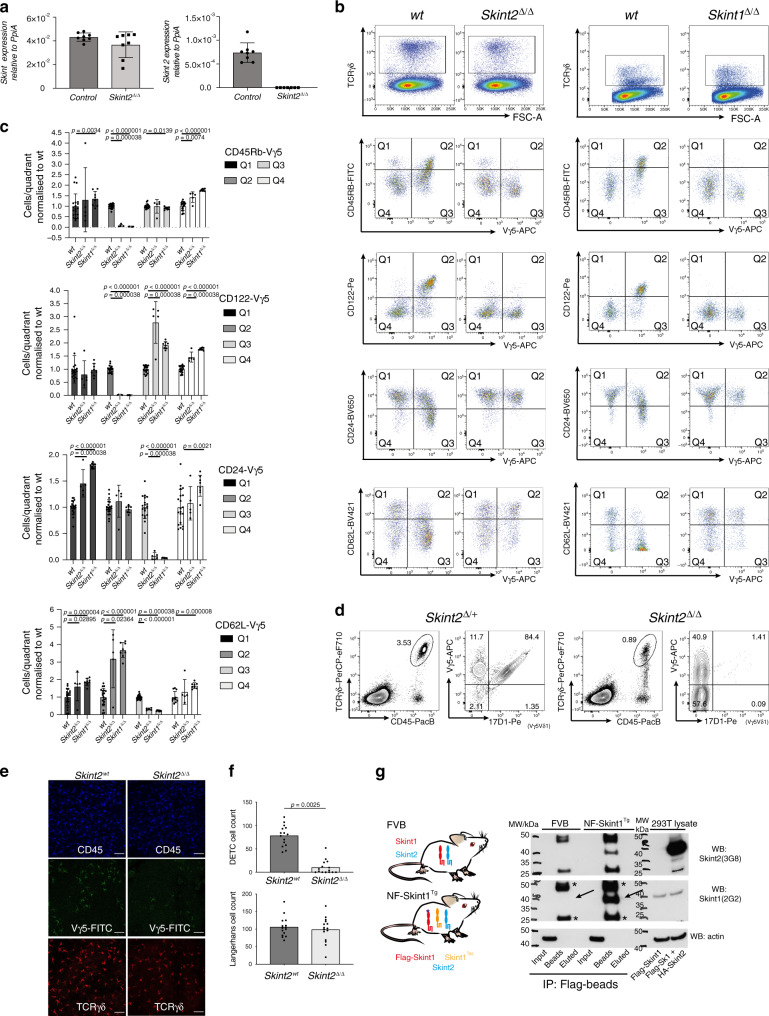
DETC development requires Skint2 and Skint1 which form heteromers. **a** qPCR analysis for *Skint1* and *Skint2* message in adult mouse ear epidermis normalised to *Ppia*. *Control*, *n* = 8,  *Skint2*^Δ/Δ^, *n* = 8. Data are mean ± SD of a representative experiment of three independent experiments. **b** Analysis of E16.5 thymocytes in WT, *Skint2*^Δ/Δ^ and *Skint1*^Δ/Δ^ animals, gated on live γδ T cells (top panel). Thymic γδ cells (gate top row) were assessed for CD45RB, CD122, CD24 and CD62L and expression of the Vγ5 TCR. Left panel: *WT* vs. *Skint2*^Δ/Δ^, right panel: *WT* vs. *Skint1*^Δ/Δ^. **c** Quantification of cell populations in quadrants Q1 to Q4 as indicated in (**b**), normalised to mean of wt = 1 for each quadrant, mean ± SD. WT *n* = 20; *Skint2*^Δ/Δ^ *n* = 5; *Skint1*^Δ/Δ^ *n* = 8 (two-tailed Man–Whitney analysis). **d** FACS analysis of ear epidermis in control (*Skint2*^Δ/+^) and *Skint2*^Δ/Δ^ mice. CD45^+^, TCRγδ^+^ cells (gate left panel) were stained for presence of Vγ5Vδ1^+^ DETC (stained by Vγ5 and 17D1 antibody, right panel). **e** Microscopy images of adult mouse ear epidermal sheets in control and *Skint2*^Δ/Δ^ mice. Comparison of DETC stained for CD45 (top: blue) and Vγ5^+^ (middle: green) γδ-TCR(GL3^+^) cells (bottom: red). Scale bar 50 µm. **f** Quantification of DETC and Langerhans cell numbers from microscopy images, *n* = 15 for each genotype (data are from three independent experiments, two-tailed Man–Whitney analysis). **g** Immunoprecipitation of Flag-tagged Skint1 from FVB or NF-Skint1^Tg^ animals. Left: scheme of FVB mice expressing Skint1 and Skint2. Scheme: Top: wt FVB mice express endogenous, untagged Skint1 and Skint2; bottom: NF-Skint1^Tg^ animals express a Flag-tagged Skint1 and untagged Skint2 on the Skint1^Tac^ background. Right: Immunoprecipitation with anti-Flag antibody from lysates of pooled thymi of FVB and NF-Skint1^Tg^ animals (*n*_FVB_ = 12, *n*_NFSkint1Tg_ = 22, 1 experiment). Expression control in 293 lysates transduced with either Flag-Skint1 alone or Flag-Skint1 & HA-Skint2 constructs. Long arrows: Skint1 band, asterisks: non-specific bands reflecting anti-FLAG Ig chain detection. Full scans are provided in Supplementary Fig. 7.

The normal, intrathymic development of Vγ5Vδ1^+^ DETC progenitors depends on *Skint1*, as judged by severe DETC depletion in *Skint1* hypomorphic (FVB.Taconic), *Skint1-*deficient (*Skint1*^Δ/Δ^) [Δ denotes internal deletion] or *Skint* locus deficient mice^17,29,30^. To ask whether DETC development depends on at least one other *Skint* gene, we used CRISPR to target *Skint2*, which seems evolutionarily conserved across rodents possessing DETC^31^. To disrupt *Skint2*, we introduced LoxP (fl) sites flanking the first and fifth protein-coding exons (exons 2 and 6). However, as is common in CRISPR strategies, a collateral outcome was an internal deletion spanning those exons (Supplementary Fig 1a). Those *Skint2*^Δ/Δ^ mice showed no *Skint2* mRNA expression in ear skin (Fig. 1a) or elsewhere, whereas wild-type (wt) *Skint1* mRNA levels were sustained.

Vγ5Vδ1^+^ DETC progenitors in the fetal thymus of ~E15.5 wt mice show *Skint1*-dependent selective maturation, as indicated by CD45RB and CD122 upregulation and CD24 and CD62L downregulation^30^. Conspicuously, Vγ5Vδ1^+^ DETC progenitors in *Skint2*^Δ/Δ^ mice phenocopied those in *Skint1* hypomorphs^30^ and *Skint1*^Δ/Δ^ animals^29^, failing to mature relative to co-examined wild-type (wt) controls, but showing compensatory increases in immature CD45RB^lo^, CD122^lo^, CD24^hi^ and CD62L^+^ cells (Fig. 1b, c). Unsurprisingly, this maturation defect resulted in almost complete loss of mature DETC expressing the 17D1 epitope displayed by the Vγ5Vδ1 DETC TCR (Fig. 1d–f; Fig Supplementary Fig. 1b). The so-called DETC-replacement cells were TCRγδ^+^, demonstrating that *Skint2* deficiency did not cause pan-γδ deficiency (Fig. 1d, e). Moreover, although they completely lacked 17D1^+^ DETC, some *Skint2*^Δ/Δ^ mice harboured Vγ5^+^ DETC-replacements (Fig. 1d–f) although their TCR expression was somewhat lower than wt Vγ5^+^ DETC, symptomatic of defective selection^18,30,32^ (Fig. 1d, e). By contrast to the dramatic change in the DETC compartment, *Skint2*^Δ/Δ^ mice showed a largely unchanged representation of MHC-class II^+^-Langerhans cells with which DETC share the epidermis (Fig. 1f; Supplementary Fig. 1b). The significance of these various phenotypic patterns notwithstanding, DETC and LC counts showed some inter-individual variation (Fig. 1f), indicative of the cells’ multifactorial regulation, although there was no evident contribution of sexual dimorphism (Supplementary Fig. 1c).

The cells with a TCR most closely related to Vγ5Vδ1^+^ DETC are uterine and lung γδ T cells expressing Vγ6 paired with a Vδ1 chain identical to that in DETC. In the absence of a generally available Vγ6-specific antibody, such cells were identified as TCRγδ^+^Vγ1^−^Vγ4^−^Vγ5^−^, and in *Skint2*^Δ/Δ^ mice such cells were largely unaffected (Supplementary Fig. 1d,e), again phenocopying *Skint1* hypomorphs^30^. Collectively, these genetic data show that *Skint2* as well as *Skint1* is critically required for the specific maturation of Vγ5Vδ1^+^ DETC progenitors, supporting the hypothesis that discrete γδ T-cell compartments are naturally regulated by Btnl heteromers.

Indeed, the capacity of Skint1 and Skint2 to form physical complexes in vitro and in vivo was validated when anti-Skint1 immunoprecipitates from 293T cells transfected with N-terminal Flag-tagged Skint1 and HA-tagged Skint2 were shown to contain both Skint1 and Skint2, as detected by western blot (Supplementary Fig. 1f). Moreover, anti-Skint1 and anti-Skint2 antibodies could detect Skint1 and Skint2, respectively, in western blots of anti-Flag immunoprecipitates from thymi of transgenic mice expressing an N-terminal Flag-tagged Skint1 construct (NF-Skint1^Tg^)^32^, but not from non-transgenic FVB mice (Fig. 1g; long arrows). Note that the detection of anti-Flag antibody chains in the FVB lysates (Fig. 1g; asterisks) validated protein loading. Moreover, the specificity of Skint1 and Skint2 detection in the immunoprecipitates was verified by the failure to detect actin in anti-Flag immunoprecipitates, despite its detection in total input protein (Fig. 1g, lowest panel). The failure to detect Skint1 or Skint2 in total input protein is consistent with their very low levels of protein expression^32^. The inefficiency of Skint1/Skint2 elution from the beads seemingly reflects a greater affinity of the anti-Flag antibody for Flag-tagged Skint proteins versus Flag peptide. This notwithstanding, the data show an evident capacity of Skint1 and Skint2 to associate in cell lines and in primary mouse tissue.

### *Btnl* genes exert hierarchical regulation of Vγ7^+^ IEL

**Fig. 2:**
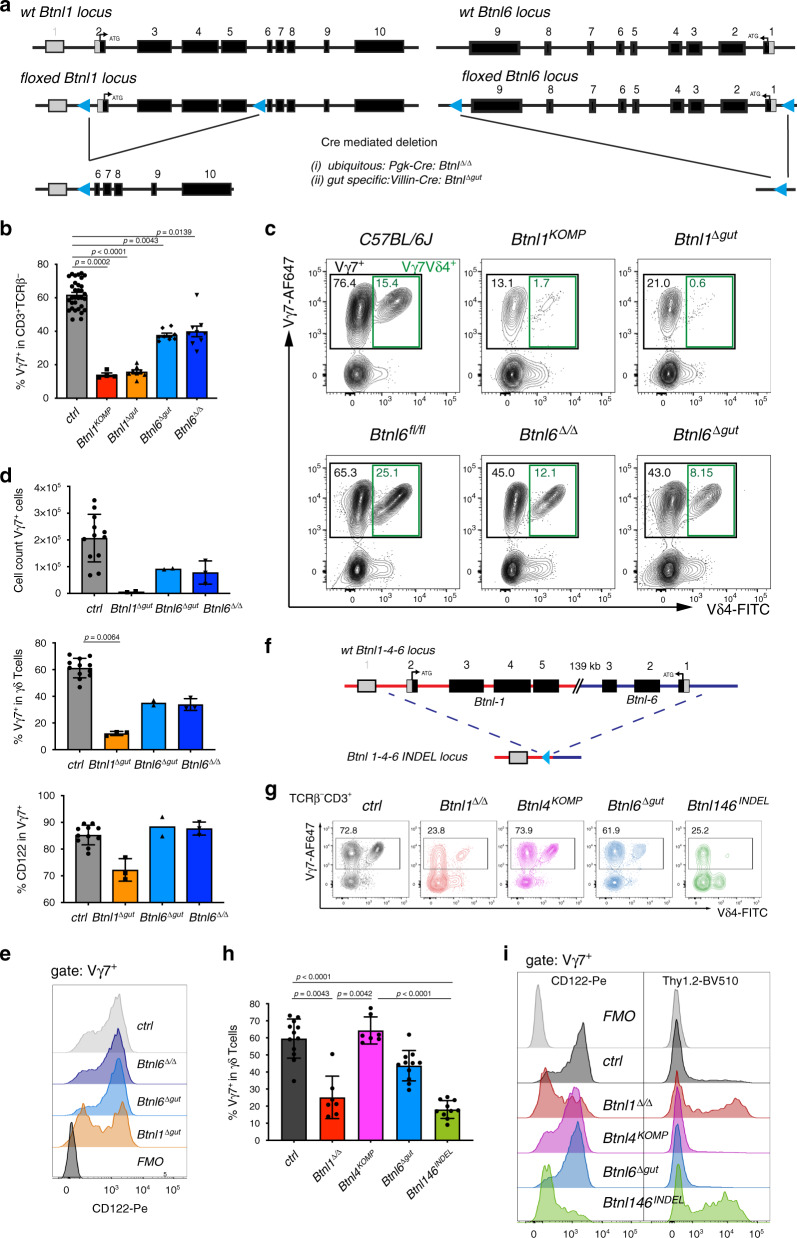
The intestinal IEL compartment is shaped by expression of distinct Btnl molecules. **a** Targeting strategy to generate conditional and constitutive *Btnl1* and *Btnl6* knockout mice. Depending on the Cre-transgenic strain used (i/ii) resulting animals are either ubiquitously deleted for the respective Btnl gene (Δ/Δ), or harbour a tissue-specific deletion (Δgut). Black: translated exons, grey: untranslated regions, blue triangles: loxP sites. **b** Quantification of Vγ7^+^ IEL (black gate in **c**) in *Btnl1* and Btnl6-deficient strains. Data are mean ± SEM of ≥2 independent experiments. *n*_ctrl_: 32, *n*_Btnl1-KOMP_: 4, *n*_Btnl1Δgut_: 8, *n*_Btnl6Δgut_: 8, *n*_Btnl6ΔΔ_: 9. Statistical analysis: Kruskal–Wallace and Dunn’s multiple comparison analysis. **c** FACS profiles of IEL preparations from animals of indicated genotypes, gated on TCRγδ^+^ cells. The label *Btnl1*^*Komp*^ indicates germline *Btnl1*^*KO*^ animals generated by the IMPC that have been described^16^. Black gate: all Vγ7^+^ cells, green gate: Vγ7^+^Vδ4^+^ cells. **d** Quantification of IEL: Vγ7^+^ IEL numbers (top), %Vγ7^+^ IEL (middle) and %CD122^+^ cells (bottom) in animals of indicated genotypes. Data are mean ± SD of a representative experiment. Top panel: *n*_ctrl_: 12, *n*_Btnl1_: 2, *n*_Btnl6Δgut_: 2, *n*_Btnl6Δ/Δ_: 3. Statistical analysis: Kruskal–Wallace & Dunn’s multiple comparison. Middle and bottom panel: *n*_ctrl_: 12, *n*_Btnl1Δgut_: 3, *n*_Btnl6Δgut_: 2, *n*_Btnl6Δ/Δ_: 3. Statistical analysis: Kruskal–Wallace & Dunn’s multiple comparison. **e** Histogram for surface expression of CD122 in Vγ7^+^ IEL from animals of the indicated genotypes. **f** Scheme depicting the strategy to generate *Btnl146*^*Indel*^ mice. Short guide RNAs flanking the 5′ region of *Btnl1* and 5′ region of *Btnl6* were injected with HDR templates. Due to the nature of CRISPR/Cas9 the intervening region was excised and a *Btnl146*^*INDEL*^ mouse lacking the *Btnl1-4-6* locus was created. Blue triangle: loxP site that was inserted due to the nature of the HDR template (see Methods). **g** FACS analysis of TCRβ^−^CD3^+^ IEL in *Btnl1/4/6*-KO and *Btnl146*^*INDEL*^ mice. Colours correspond to coloured bar graphs in (**h**) and (**i**). **h** Quantification of Vγ7^+^ IEL depicted in (**g**). Data are mean ± SEM of three independent experiments. *n*_Ctrl_: 12, *n*_Btnll1Δ/Δ_: 7, *n*_Btnl4KOMP_: 7, *n*_Btnl6Δgut_: 11, *n*_Btnl146Indel_: 10. Statistical analysis: Kruskal–Wallace & Dunn’s multiple comparison. **i** Surface expression of CD122 (left) and Thy1.2 (right) in Vγ7^+^ IEL from animals of indicated genotypes.

Small intestinal villus enterocytes express *Btnl1*, *Btnl4* and *Btnl6* genes^16,33,34^. Whereas *Btnl1*-deficient mice were substantially depleted of signature Vγ7^+^ intestinal IEL, *Btnl4* deficiency had no obvious effect^16^. Therefore, to test whether the heteromeric model also applied to the gut, we generated mice lacking *Btnl6*, which encodes a protein that can collaborate with Btnl1 to stimulate mature Vγ7^+^ intestinal IEL^16^. To this end, we introduced loxP sites on either side of the 9-exon gene (Fig. 2a, right panel; *Btnl6*^*fl/fl*^ mice). In parallel, to complement the *Btnl1*^*KO*^ strain previously obtained from the International Knockout Mouse Consortium (*Btnl1*^*KOMP*^)^16^, we generated a floxed allele of *Btnl1* with LoxP sites flanking the first four coding exons (exons 2*–*5) (Fig. 2a, left panel). A constitutive, universal knockout of *Btnl6* (*Btnl6*^Δ/Δ^ mice) was generated by crossing floxed *Btnl6* with *Pgk-Cre* mice^35^, while intestinal epithelial cell (IEC)-specific knockouts of *Btnl6* (*Btnl6*^Δ*gut*^ mice) and *Btnl1* (*Btnl1*^Δ*gut*^ mice) were generated by crossing the floxed mice to *Villin-Cre* mice^36^ (Fig. 2a). The veracity of the different mutant mouse strains was evident from quantitative RT-PCR of *Btnl1*, *Btnl4* and *Btnl6* expression, and histologic RNAScope analysis of *Btnl1* and *Btnl6* (Supplementary Fig. 2a,b).

The intestinal IEL compartment of adult *Btnl1*^Δ*gut*^ mice strikingly phenocopied the complete *Btnl1* knockout, displaying substantial and significant reductions of Vγ7^+^ cells, and of Vγ7Vδ4^+^ cells that we previously showed to be particularly affected by *Btnl1* deficiency^16^: hence, the profound γδ-regulatory impact of Btnl1 seems attributable exclusively to IEC (Fig. 2b, c). Most unexpectedly, however, *Btnl6*^Δ/Δ^ and *Btnl6*^Δ*gut*^ mice showed an overtly intermediate phenotype, with Vγ7^+^ IEL and Vγ7Vδ4^+^ IEL significantly reduced relative to either C57BL/6 or *Btnl6*^*fl/fl*^ mice, as measured by a 33–50% decrease in their percentage representation among gut γδ cells or by a 2-fold drop in absolute numbers of Vγ7^+^ IEL, by contrast to the cells’ almost complete loss from *Btnl1*^Δ*gut*^ mice (Fig. 2b–d).

The few residual IEL in *Btnl1*^Δ*gut*^ mice showed major dysregulation of the signature Vγ7^+^ IEL phenotype, with many cells displaying low expression of CD122 (the IL-15R chain) and high Thy1 (CD90) expression (Fig. 2d, e; Supplementary Fig. 2c). Strikingly, such dysregulation was not true for *Btnl6*^Δ*gut*^ or *Btnl6*^Δ/Δ^ mice, in which residual IEL showed comparable phenotypes to controls (Fig. 2d, e; Supplementary Fig. 2c). Furthermore, whereas the Vγ7Vδ4 TCR mean fluoresecence intensity (MFI) was somewhat lower in *Btnl1*-deficient mice, consistent with defective selection^16^, it was unaltered in *Btnl6*^Δ*gut*^ and *Btnl6*^Δ/Δ^ mice (see Fig. 2c). Hence, *Btnl1* and *Btnl6* differentially affected Vγ7^+^ IEL development, with *Btnl6* required for the normal size of the IEL compartment, but not for the acquisition of the signature phenotype by residual Vγ7^+^ IEL. Moreover, the unique phenotype of *Btnl6*^Δ/Δ^ mice was specific in that myriad immune subsets in the spleen were unaltered relative to controls (Supplementary Fig. 2d; Supplementary Table 1), as was reported for *Btnl1*^*−/−*^ mice^16^.

To test whether those Vγ7^+^ IEL that developed seemingly normally in *Btnl6*-deficient mice were *Btnl-*dependent, we generated mice lacking all three intestinal epithelial Btnls, by targeting sites upstream of the initiator ATG codons in *Btnl1* and *Btnl6*, respectively (note that those genes are transcribed in head-to-head orientation), thereby deleting over 130 kb in between, including the *Btnl4* gene (Fig. 2f). The resultant (*Btnl146*^*INDEL*^) mice expressed no detectable *Btnl1, Btnl4* or *Btnl6* transcripts, and there was also reduced expression of the *Btnl2* gene that immediately flanks the deletion. Conversely, *Psmb9*, which is more distal to the recombination point was unaffected (Supplementary Fig. 2e).

When *Btnl146*^*INDEL*^ mice were contemporaneously compared with *Btnl1*^Δ/Δ^, *Btnl6*^Δ*gut*^ and *Btnl4-*deleted mice (*Btnl4*^*KOMP*^) that we previously characterised^16^, it was clear that *Btnl146*^*INDEL*^ mice (colour-coded green in Fig. 2g–i) largely phenocopied the near-ablation of Vγ7^+^ IEL in *Btnl1*-deficient mice (Fig. 2g, h). In both cases, residual Vγ7^+^ cells showed reduced Vγ7 and Vδ4 TCR MFI and failed to upregulate CD122 or downregulate Thy1, by comparison to IEL in control, *Btnl4*^*KOMP*^ or *Btnl6-*deficient strains (Fig. 2g, i). When we integrated data from large numbers of mice of the different strains described, it became clear that the strains’ respective Vγ7^+^ IEL compartments were consistent and stable over time for >120 days (Supplementary Fig. 2f).

In sum, further support for the heteromer hypothesis was provided by the developmental dependence of approximately one-third to one-half of intestinal Vγ7^+^ IEL on *Btnl6* as well as *Btnl1*. Nonetheless, an unanticipated nuance was introduced in that there was a hierarchy of *Btnl* regulation, with Vγ7 IEL numbers depending almost completely on *Btnl1*, partially on *Btnl6*, and not on *Btnl4*, while the signature phenotypes of Vγ7^+^ IEL present in the different strains were largely dependent on *Btnl1*, but independent of either *Btnl6* or *Btnl4*.

### *Btnl6* deficiency alters Vδ gene usage

**Fig. 3:**
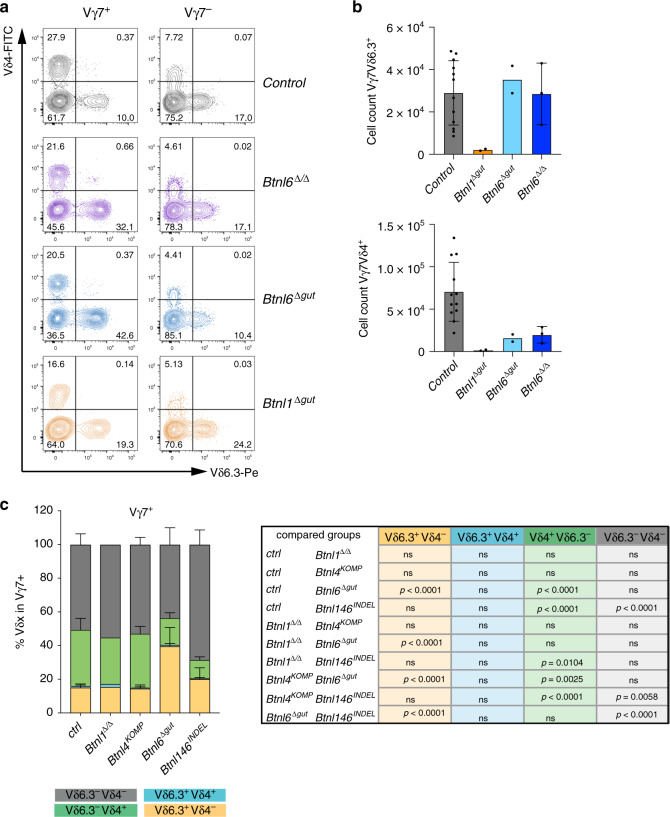
*Btnl6* deficiency alters Vδ gene usage. **a** FACS plots of TCRδ chain usage in animals of indicated genotypes. The Vδ4 and Vδ6.3 chains are plotted against each other in Vγ7^+^ IEL (left column) and Vγ7^−^ cells (right column). **b** Quantification of Vγ7^+^Vδ6.3^+^ (top) and Vγ7^+^Vδ4^+^ cell numbers (bottom), in animals of indicated genotypes. *n*_ctrl_: 12, *n*_Btnl1_: 2, *n*_Btnl6Δgut_: 2, *n*_Btnl6Δ/Δ_: 3. Data are mean ± SD of a representative experiment. **c** Quantification of Vδ-chain usage in Vγ7^+^ IEL as depicted in quadrants (**a**) in animals of indicated genotypes *n*_ctrl_: 7, *n*_Btnl1_: 2, *n*_Btnl4KOMP_: 3, *n*_Btnl6Δgut_: 5, *n*_Btnl146Indel_: 4. Statistical analysis: two-way ANOVA & Tukey’s multiple comparison post test. Data are mean ± SD of a representative experiment.

Although Vγ7 usage denotes the signature intestinal γδ IEL compartment, Vδ usage is also limited to some degree, with Vδ4 (encoded by the *Trdv2-2* gene) and Vδ7 predominating, whereas ≤10–15% of Vγ7^+^ cells express TCR Vδ6.3 (encoded by identical *Trdv6D-1* and *Trdv6N-1* genes) (Fig. 3a)^19^. Conversely, slightly more Vγ7^(−)^ IEL in wt mice expressed Vδ6.3, although the TCR MFI was lower vis-à-vis Vγ7^+^Vδ6.3^+^ cells, typical of unselected cells (Fig. 3a, top panels).

By contrast, Vδ6.3^+^ cells with high TCR MFI accounted for >30% of residual Vγ7^+^ IEL in both *Btnl6*-mutant strains, a highly significant difference from controls (Fig. 3a–c, Supplementary Fig. 3). This unanticipated finding reflected the fact that Vγ7^+^Vδ6.3^+^ IEL numbers were essentially unaltered in *Btnl6*^Δ*gut*^ and *Btnl6*^Δ/Δ^ mice versus wt mice, despite total Vγ7^+^ IEL being reduced by ~2-fold (above) (Fig. 3b). Although some *Btnl1*^Δ*gut*^ mice showed small increases in the percentage of Vδ6.3^+^ cells among Vγ7^+^ IEL (Fig. 3a, bottom plots; Supplementary Fig. 3), the absolute numbers of Vγ7^+^ IEL in this strain were so neglible as to make such comparisons somewhat unreliable (Fig. 3b). Indeed, the very few residual Vγ7^+^ IEL in *Btnl146*^*INDEL*^ mice showed no significant increases in Vδ6.3 representation, although there was some reduction in Vδ4 usage (Fig. 3c, Supplementary Fig. 3a). In sum, Vγ7^+^Vδ6.3^+^ IEL showed essentially no requirement for *Btnl6*, by contrast to their dependence on *Btnl1*. We therefore investigated whether Vγ7^+^Vδ6.3^+^ might be regulated by a Btnl1 + Btnl4 heteromer.

### Vγ7^+^ IEL respond to different Btnl combinations

**Fig. 4:**
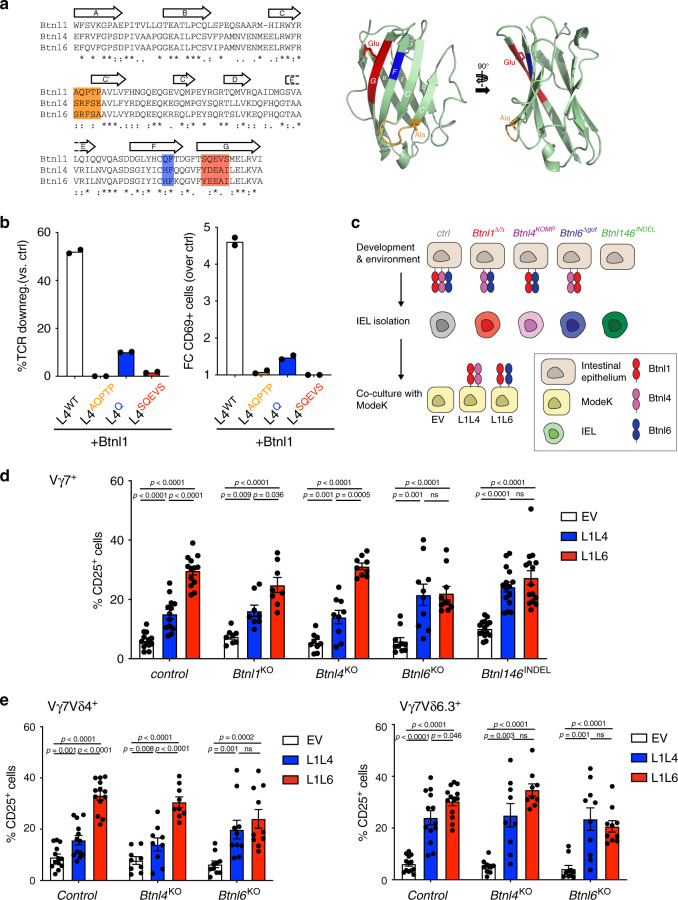
Vγ7^+^ IEL respond to different Btnl pairings. **a** Left: Alignment of the IgV-domain sequences of Btnl1, Btnl4 and Btnl6. Canonical Ig-fold β-strands [A, B, C, C′, C″, D, E, F, G] are indicated with arrows. CFG face motifs previously shown in Btnl6 to be critical for the response of Vγ7^+^ cells^19^ are highlighted in orange [AQPTP/SRFSE/SRFSA], blue [QF/HF/HF] and red [SQEVS/YDEAI/YEEAI]. Right: Cartoon representation of the IgV-domain of Btnl6, derived with 3D-JIGSAW from the crystal structure of BTN3A1 (PDB accession code 4F80), with the same annotation as in (**a**). Side chains are displayed for the two residues that differ in the CFG face motifs of Btnl6 versus Btnl4 (Ala versus Glu, Glu versus Asp). **b** TCR downregulation (left) and CD69 upregulation (right) by J76 cells expressing a Vγ7Vδ4 TCR and co-cultured with 293T transiently transfected with Btnl1 in combination with Btnl4 wild-type (L4^WT^) or mutated in the CFG region as indicated on the X-axis. Results are normalised to 293T transfected with empty vector (EV). Data are represented as mean ± SD of duplicate co-cultures, representative of *n* = 2 independent experiments. FC, fold change. **c** Experimental setup to analyse IEL from various KO strains in co-cultures with MODE-K cells overexpressing either Btnl1 and Btnl4 (L1L4) or Btnl1 and Btnl6 (L1L6). IELs are isolated from indicated mouse strains which can display distinct combinations of Btnl molecules on the epithelial surface during development. Following isolation, IEL were co-cultured o.n. with MODE-K cells displaying either Btnl1+4 or Btnl1+6 on their surface. MODE-K cells transduced with empty vector (EV) were used as control. **d** IEL response to MODE-K cells expressing different Btnl dimers (L1L4 or L1L6) was measured by analysing CD25^+^ cells gated on Vγ7^+^ cells in animals of indicated genotypes. Data are mean ± SEM of five independent experiments, *n*_ctrl_: 13, *n*_Btnl1KO_: 8, *n*_Btnl4KO_: 9, *n*_Btnl6KO_: 10, *n*_Btnl146Indel_: 15. Statistical analysis: two-way ANOVA & Tukey’s multiple comparison post test. **e** IEL response to MODE-K cells expressing different Btnl dimers (L1L4 or L1L6) was measured as %CD25^+^ cells and further gated on Vγ7^+^Vδ4^+^ (left) or Vγ7^+^Vδ6.3^+^ (right) cells in animals of indicated genotypes. Data are mean ± SEM of five independent experiments, *n*_ctrl_: 13, *n*_Btnl4KO_: 9, *n*_Btnl6KO_: 10. Statistical analysis: two-way ANOVA & Tukey’s multiple comparison post test.

Biochemical and molecular evidence has shown that Btnl1 + Btnl6 function is mediated by Btnl6 engaging Vγ7, while Btnl1 acts as a critical chaperone^19,37^. Btnl4 has near-identity to Btnl6 in the region (CFG) that engages Vγ7 and both are diverged from Btnl1 (Fig. 4a, colour-coded orange, blue and red). To interrogate whether Btnl4 might substitute for Btnl6, we subjected primary IEL to co-culture with the MODE-K enterocyte cell line expressing either Btnl1 + Btnl6 (L1L6) or Btnl1 + Btnl4 (L1L4). The former pairing induced TCR downregulation and CD25 upregulation in Vγ7^+^ IEL from wt mice^16,19^ (Supplementary Fig. 3b; top row, right panel), relative to which a significant albeit reduced effect was also induced by Btnl1 + Btnl4 (Supplementary Fig. 3b; top row, centre panel).

This response was further investigated by co-expressing *Btnl1* in 293T cells with *Btnl4* alleles mutated in each of three regions whose counterparts in *Btnl6* are implicated in Vγ7 engagement (Fig. 4a)^19^. Specifically, the *Btnl4* sequences were replaced by counterparts from *Btnl1*, in one case via the substitution of a single amino acid. Those mutations essentially ablated the TCR downregulation and CD69 upregulation ordinarily induced by Btnl1 + Btnl4 in a human T-cell line, J76, expressing a monoclonal Vγ7Vδ4 TCR (Fig. 4b). Thus, Btnl4 can phenocopy Btnl6 in co-operating with Btnl1 to regulate Vγ7^+^ IEL, albeit that no IEL depend on *Btnl4* for their maturation, as shown above.

To further examine responses to Btnl1 + 4 versus Btnl1 + 6, we examined CD25 upregulation by primary Vγ7^+^ IEL from different *Btnl* mutant strains (Fig. 4c). Vγ7^+^ IEL from wt mice were phenocopied by Vγ7^+^ IEL from *Btnl4*^−/−^ mice, and by those few residual Vγ7^+^ cells in *Btnl1*^−/−^ mice in responding better to MODE-K cells expressing Btnl1 + 6 compared with those expressing Btnl1 + 4. However, this was not true for Vγ7^+^ IEL from *Btnl6-*deficient mice, which responded comparably or better to Btnl1 + 4 (Fig. 4d; Supplementary Fig. 3b). Likewise, the very few residual Vγ7^+^ IEL in *Btnl146*^*INDEL*^ mice showed comparable responses to Btnl1 + 4 and Btnl1 + 6 (Fig. 4d; Supplementary Fig. 3b).

When the responding Vγ7^+^ IEL were further scrutinised, it became clear that in wt and *Btnl4*^−/−^ mice, the striking discrimination between Btnl1 + 6 and Btnl1 + 4 largely reflected the responses of Vγ7Vδ4^+^ and Vγ7Vδ4^−^Vδ6.3^−^ IEL (Fig. 4e, left panel; Supplementary Fig. 3c, top two rows; Supplementary Fig. 3d). By contrast, largely comparable responses to Btnl1 + 6 and Btnl1 + 4, respectively, were made by Vγ7Vδ6.3^+^ IEL (Fig. 4e, right panel; Supplementary Fig. 3c, bottom two rows), which are over-represented in *Btnl6*^−/−^ mice in which *Btnl-*dependent selection would of necessity be driven by Btnl1 + 4. In fact, Vγ7Vδ6.3^+^ IEL, Vγ7Vδ4^+^ IEL and Vγ7Vδ4^−^Vδ6.3^−^ IEL (which are mostly Vδ7^+^)^19^ from *Btnl6*^−/−^ mice all showed relatively strong responses to Btnl1 + 4 (Fig. 4e; Supplementary Fig. 3b,c), possibly consistent with their having been selectively expanded and matured by some combination of Btnl4 and Btnl1.

To further investigate Vγ7^+^ IEL regulation by Btnl1, Btnl4 and Btnl6, we expressed each separately and in combination in 293T cells. Surface display of Btnl6 was highly inefficient, but was rescued by co-expression of Btnl1 (Supplementary Fig. 4a). Conversely, Btnl4 alone could travel to the cell surface (Supplementary Fig. 4a): hence, Btnl6 and Btnl4 are not strictly comparable. Vγ7^+^ J76 transductants (see above) were strongly stimulated by cells co-expressing Btnl1 + 6 but not by cells expressing Btnl6 alone, nor by an admixture (Btnl1/Btnl6 sep) of cells expressing Btnl6 with cells expressing Btnl1 (Supplementary Fig. 4b, red). Conversely, 293T cells transduced with Btnl4 alone showed some capacity to stimulate Vγ7^+^ J76 cells, although this was clearly increased by co-expressing Btnl1, but not by stimulating with an admixture of cells separately expressing Btnl4 and Btnl1 (Supplementary Fig. 4b, blue). These data evoke the activity of human BTNL3, a human Vγ4^+^-TCR ligand, which alone can provoke human Vγ4^+^-TCR downregulation, but whose effects are greatly amplified by BTNL8 co-expression^19^.

In sum, Btnl4 is evidently not required for Vγ7^+^ IEL selection, but its capacity to stimulate Vγ7^+^ IEL in vitro likely explains its capacity to select IEL, primarily Vγ7^+^Vδ6.3^+^, in *Btnl6*-deficient mice. The starkly different phenotypes of *Btnl6*-deficient and *Btnl1-*deficient mice, argues that any Btnl4*-*intrinsic capacity to select Vγ7^+^ IEL relies in vivo on co-expression with *Btnl1*. Added to this, our data show that the signature preferential responses of Vγ7Vδ4^+^ cells to Btnl1 + 6 was seen only in cells from mice in which Btnl6 was expressed. This conditioning might be enforced during developmental selection, and/or be maintained in the steady-state by Btnl heteromers expressed in epithelial cells that juxtapose mature IEL. However, there has not hithtero been formal evidence of a maintenance function for *Btnl* genes, beyond their roles in selection. We therefore investigated this by use of conditional knockout mice.

### Phenotypic maintenance by *Btnl1* and *Btnl6*

**Fig. 5:**
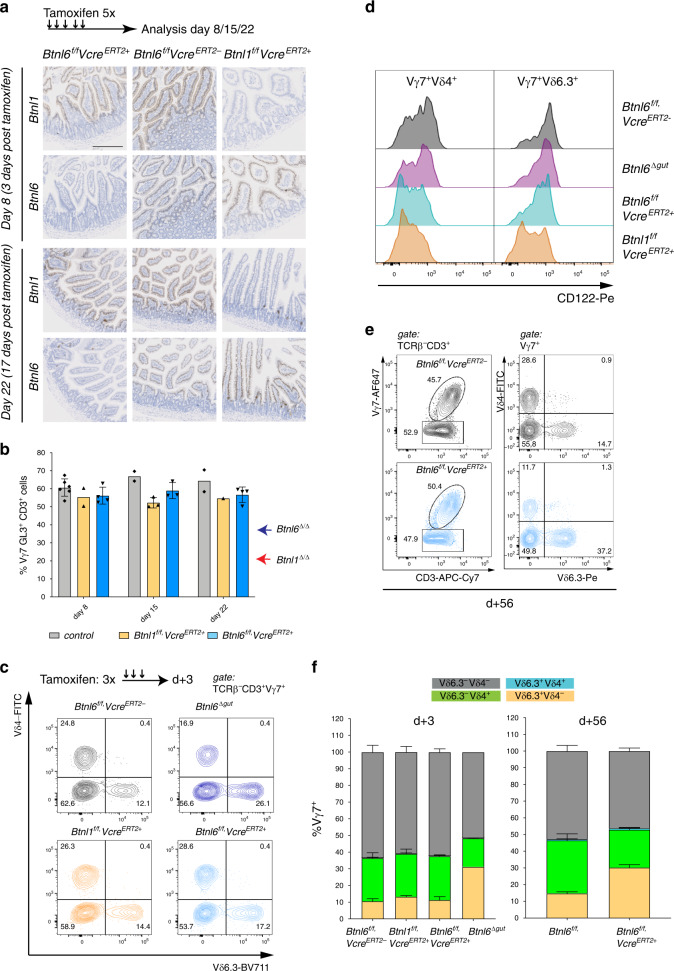
Depletion of individual *Btnl* genes does not impact Vγ7 IEL numbers but differentially affects CD122 expression. **a** Top: Experimental scheme to analyse the effect of *Btnl1* and *Btnl6* tamoxifen-mediated depletion at different timepoints. Bottom: RNAscope analysis for *Btnl1* and *Btnl6* in animals of indicated genotypes at 3 or 17 days post tamoxifen administration. Data are representative micrographs from one time course experiment with numbers of gut sections stained per genotype as: day 8: *n*_ctrl_: 4, *n*_Btnl1f/f-VcreERT2+_: 2, *n*_Btnl6f/f-VcreERT2+_: 4, day 22: *n*_ctrl_: 4, *n*_Btnl1f/f-VcreERT2+_: 1, *n*_Btnl6f/f-VcreERT2+_: 2, scale bar: 200 µm. **b** Quantification of Vγ7^+^ cells at indicated timepoints post tamoxifen (red and blue side arrows denote for comparison the average percentage of Vγ7^+^ IEL in full knockout animals (see also Figs. 1–3). Data are mean ± SD, day 8: *n*_ctrl_: 6, *n*_Btnl1f/f-VcreERT2+_: 2, *n*_Btnl6f/f-VcreERT2+_: 4, day 15: *n*_ctrl_: 2, *n*_Btnl1f/f-VcreERT2+_: 3, *n*_Btnl6f/f-VcreERT2+_: 3, day 22: *n*_ctrl_: 2, *n*_Btnl1f/f-VcreERT2+_: 1, *n*_Btnl6f/f-VcreERT2+_: 4. **c** Top: Experimental scheme to analyse the effect of Btnl1 and Btnl6 tamoxifen-mediated depletion after 3 days. Bottom: Vδ-chain usage in Vγ7^+^ IEL in control (black), *Btnl1*^*f/f*^*,Villin*^*CreERT2*+^ (orange), *Btnl6*^Δ*gut*^ (purple) and *Btnl6*^*f/f*^*,Villin*^*CreERT2*+^ (light blue) animals. The Vδ4 and Vδ6.3 chain gated on Vγ7^+^ IEL are plotted against each other. **d** Histogram of surface CD122 expression in indicated subpopulations of Vγ7^+^ IEL in animals of indicated genotypes. **e** Percentage of Vγ7 cells (left) and usage of the Vδ4 and Vδ6.3 chain (right) in Vγ7^+^ IEL, 56 days after tamoxifen in *Btnl6*^*f/f*^ *VillinCre*^*ERT2−*^ (black) and *Btnl6*^*f/f*^ *VillinCre*^*ERT2*+^ (blue) animals. **f** Quantification of Vδ-chain usage in Vγ7^+^ IEL in control, *Btnl1*^*f/f*^*,Villin*^*CreERT2*+^ and *Btnl6*^*f/f*^*,Villin*^*CreERT2*+^ knockout animals, 3 (left graph) and 56 days (right) after tamoxifen administration. Mean ± SEM from two experiments per timepoint, left graph: *n*_ctrl_: 6, *n*_Btnl1f/f-VcreERT2+_: 3, *n*_Btnl6f/f-VcreERT2+_: 6, *n*_Btnl6Δgut_: 2, right graph: *n*_ctrl_: 5, *n*_Btn61f/f-VcreERT2+_: 15.

We crossed floxed *Btnl1* and *Btnl6* strains to tamoxifen-regulated *Villin-Cre* mice, in order to generate mice in which *Btnl1* and *Btnl6* were inducibly deleted in IEC. Indeed, there was sustained loss of *Btnl1* and *Btnl6* expression, as assessed by RNAscope at 8 days and 22 days following the start of 5 days’ of tamoxifen administration (Fig. 5a), whereas there was no effect of tamoxifen treatment on mice lacking the relevant *Cre* allele (Fig. 5a; middle column). Durable loss of *Btnl1* and *Btnl6* expression suggested that gene deletion had occurred in enterocyte stem cells, as reported^36^. Over a 15-day period post tamoxifen-mediated *Btnl1/Btnl6* deletion, no significant reduction was apparent in the representation of Vγ7^+^ intestinal IEL, particularly by comparison to the reduced numbers seen in constitutively deleted *Btnl6*^Δ/Δ^ mice and *Btnl1*^Δ/Δ^ mice (Fig. 5b). Thus, signature IEL could be maintained at steady-state for at least two weeks in the absence of either *Btnl1* or *Btnl6*.

Nonetheless, to investigate whether there might be more immediate effects of *Btnl1/Btnl6* deletion, we applied tamoxifen daily for 3 days, and examined IEL 3 days later (Fig. 5c). Within this short time-frame, CD122 expression was markedly reduced on a large percentage of Vγ7^+^ IEL in both *Btnl1*^*fl/fl*^*Vcre*^*ERT2*+^ and *Btnl6*^*fl/fl*^*Vcre*^*ERT2*+^ mice (Fig. 5d). While this echoed the limited expression of CD122 by residual Vγ7^+^ IEL in constitutive *Btnl1*^−/−^ mice, it seemed a priori to conflict with sustained CD122 expression in constitutive *Btnl6*^−/−^ mice (above). This conflict, however, was resolved by the finding that in mice acutely depleted of *Btnl6*, rapid CD122 downregulation was mostly limited to approximately half of Vγ7Vδ4^+^ IEL (Fig. 5d). Indeed, Vγ7Vδ6.3^+^ IEL (that are disproportionately enriched in constitutive *Btnl6*^−/−^ mice) were much less affected by acute depletion of *Btnl6* versus *Btnl1* (Fig. 5d), providing another example of the differential effects of Btnl proteins on different Vγ7^+^ IEL subsets.

In this regard, we hypothesise that Vγ7Vδ6.3^+^ IEL may have been selected on Btnl1 + 4 even in wt mice, with their CD122 expression likewise maintained by Btnl1 + 4; hence, they were essentially insensitive to acute *Btnl6* depletion, phenocopying Vγ7Vδ6.3^+^ IEL and some Vγ7Vδ4^+^ IEL in constitutive *Btnl6*^−/−^ mice. Evidence in support of this hypothesis was provided by further analysis of Vδ usage by Vγ7^+^ IEL, which was essentially unaffected at 3 days following *Btnl6* depletion, but which was significantly skewed toward Vδ6.3^+^ cells by day 56 (Fig. 5e, f). This would be consistent with natural IEL turnover favouring newly-maturing Vγ7 Vδ6.3^+^ IEL versus Vγ7Vδ4^+^ IEL, since following Btnl6 deletion, the former could more efficiently engage Btnl1 + 4.

The differential impacts of Btnl1, 4 and 6 on different IEL subsets might reflect their different spatio-temporal regulation. We therefore analysed single-cell RNA data available from studies in which distinct small intestinal populations were investigated. Consistent with our and others’ studies^34,38^, all three Btnls were restricted to enterocytes and enterocyte progenitors (Supplementary Fig. 5a), and spatially each peaked in the middle of the basal-apical villus axis (regions V2–V4) (Supplementary Fig. 5b), aligning with the distribution of γδ^+^ IEL^39^. In sum, there was no obvious difference in spatio-temporal expression that might explain the proteins’ differential effects, although there was an apparent hierarchy of RNA expression levels—Btnl1 >> Btnl6 > Btnl4—that evoked the hierarchy of the genes’ effects on Vγ7^+^ IEL.

### Response maintenance by *Btnl1* and *Btnl6*

**Fig. 6:**
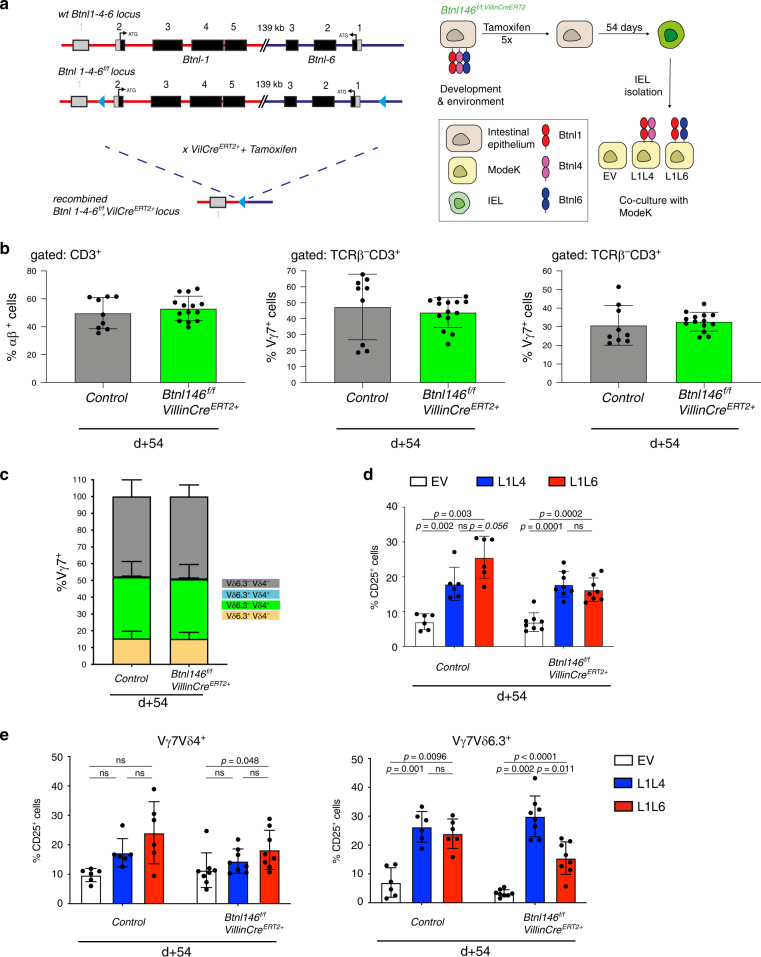
Response and maintenance by *Btnl1* and *Btnl6*. **a** Left: Targeting strategy to generate animals harbouring a floxed Btnl146 locus, which can be excised after tamoxifen administration. Right: Experimental design for IEL analysis (**c**, **d**) and co-culture experiment (**e**) following *Btnl146* locus depletion. During development Btnl molecules are expressed on the IEC and only after tamoxifen depletion Btnl expression is lost. Following loss of Btnl expression, IELs are harvested and subjected to co-cultures with MODE-K cells expressing specific Btnl combination. **b** Quantification of αβ (left), Vγ7^+^ (middle) and Vγ1^+^ (right) T cells following *Btnl146* locus depletion. *n*_ctrl_: 9, *n*_Btnl146VcreERT2+_: 14. Data are mean ± SD. **c** Quantification Vδ chain usage in Vγ7^+^ cells in animals of indicated genotypes under indicated conditions. *n*_ctrl_: 6, *n*_Btnl146VcreERT2+_: 8. Data are mean ± SD. **d** Co-culture of MODE-K cells transduced with EV, L1L4 or L16 with IEL from control, or *Btnl146*^*f/f*^*;Villin*^*CreERT2*+^ animals. Controls are pooled: *Btnl146*^*f/f*^*,Villin*^*CreERT2*+^ that did not receive tamoxifen and *Btnl146*^*f/f*^*,Villin*^*CreERT2*–^ animals that did receive Tamoxifen. Data are mean ± SD (*n*_ctrl_: 6, *n*_Btnl146VcreERT2+_: 8). Statistical analysis two-way ANOVA & Tukey’s multiple comparison post test. **e** IEL response in co-cultures of MODE-K cells transduced with EV, L1L4 or L1L6 with IEL from *control* or *Btnl146*^*f/f*^;*Villin*^*CreERT2+*^ animals that did receive tamoxifen in Vγ7Vδ4^+^(left graph) and Vγ7Vδ6.3^+^(right graph) cells. *n*_ctrl_: 6, *n*_Btnl146VcreERT2+_: 8.

To further investigate the requirement for sustained expression of *Btnl* genes, we examined IEL at 54 days after gut epithelium-specific deletion of the whole *Btnl1,4,6* locus, making comparisons with wt mice and constitutive *Btnl146*^*INDEL*^ mice (Fig. 6a). (Note, acute loss of *Btnl1,4,6* could not be examined because of variable penetrance of locus deletion until 1-month post tamoxifen treatment.) Locus loss for ~8 weeks again failed to diminish Vγ7^+^ IEL numbers (Fig. 6b, middle panel, light green bars), supporting the conclusion that steady-state maintenance of *Btnl-*selected Vγ7^+^ IEL numbers does not require sustained Btnl expression. Moreover, there was no significant increase in Vδ6.3^+^ cells consistent with there being no Btnl1 + 4 heteromers to promote their selective advantage (Fig. 6c). Interestingly, however, induced *Btnl1,4,6* locus deletion also phenocopied constitutive *Btnl146*^*INDEL*^ mice in that the capacity of co-cultured Vγ7^+^ IEL to respond preferentially to Btnl1 + 6 versus Btnl1 + 4 was lost over time (Fig. 6d). Diminished responses to Btnl1 + 6 were seen for Vγ7Vδ4^+^ IEL and particularly for Vγ7Vδ6.3^+^ cells (Fig. 6e). This provides further support for the hypothesis that *Btnl6* needs to be sustained to establish and to maintain the phenotype of cells that preferentially respond to Btnl1 + 6.

## Discussion

γδ T cells, particularly those residing within extralymphoid tissues, have been increasingly implicated in the regulation of tissue maintenance and protection against cancer^39–44^. Nonetheless, the cells’ biologies remain poorly elucidated. Germane to this, a substantive advance was made by the discovery that different compartments of mouse and human γδ T cells are critically and specifically regulated by butyrophilin and butyrophilin-like (Btnl) proteins. Moreover, recent cell biological, molecular and biochemical data fuelled the hypothesis that the active forms of Btnl proteins may be heterodimers, although there was heretofore no formal evidence supporting this in vivo. This study now provides genetic evidence for the importance of Skint/Btnl proteins functioning collaboratively, as would be the case for heteromers. In addition, our approach has revealed some surprising findings that emphasise the importance of genetics in understanding cell regulatory mechanisms.

Thus, signature murine skin γδ IEL are shown to depend upon *Skint1* + *Skint2* and the normal intestinal γδ IEL compartment shown to depend upon *Btnl1* + *Btnl6*. However, whereas *Btnl1*^Δ/Δ^ mice lacked the great majority of Vγ7^+^ IEL, ~50% were retained in different strains of *Btnl6*-deficient mice. In seeking to understand this unanticipated hierarchy of Btnl proteins, we identified a potential of Btnl4 to substitute for Btnl6. However, whereas Vγ7^+^ IEL from wt mice ordinarily responded better in vitro to Btnl1 + Btnl6 versus Btnl1 + Btnl4, this was not so in *Btnl6*-deficient mice wherein the compartment of mature Vγ7Vδ6.3^+^ and Vγ7Vδ4^+^ IEL, that was presumably selected by Btnl1 + Btnl4, responded comparably well to Btnl1 + Btnl4.

The CD122^hi^ phenotype of most Vγ7^+^ IEL was reduced when *Btnl1* was acutely depleted, providing formal evidence that sustained expression of a *Btnl* gene-product is required to maintain the signature status of the wt γδ IEL compartment. By contrast, only a fraction of Vγ7^+^ IEL showed CD122 downregulation when *Btnl6* was acutely depleted. Moroever, the unaffected cells were enriched in Vδ6.3^+^ cells, phenocopying the repertoire composition in constitutive *Btnl6*-deficient mice. These data are consistent with the hypothesis that whereas Btnl4 is not required for the selection and/or maintenance of any Vγ7^+^ IEL^16^, some IEL in wt mice have naturally selected on Btnl1 + Btnl4 while others selected on Btnl1 + Btnl6. Indeed, we propose that discrete Btnl heteromers ordinarily select those cells that respond most strongly to them and/or that they condition the responses of the cells they select. Thereafter, the Btnl heteromer on which the cells are selected is required to maintain the cells’ signature phenotype. In sum, Btnl proteins operate in different combinations (i.e. show combinatorial diversity) in refining and regulating the composition of IEL compartments.

The biophysical basis for the preference of some cells for Btnl1 + Btnl6 versus Btnl1 + Btnl4 is unresolved. Btnl6 and Btnl1 physically associate, either directly or via an intermediate, and in this complex Btnl6 seemingly interacts directly with Vγ7^37^. Although there is currently less evidence available for the direct interaction of Btnl4 with Btnl1, it is likely that the two associate given the Btnl1-dependence of essentially all Vγ7^+^ IEL, and the capacity of Btnl1 co-expression to greatly increase the impact of Btnl4 on Vγ7^+^ IEL. In this regard, the association of BTN2A1 and BTN3A1, which are jointly required to regulate human Vγ9Vδ2 cells, only became evident after chemical cross-linking^45^. Nonetheless, some capacity of some mouse or human Btnl proteins (e.g. Btnl4; BTNL3), when over-expressed, to traffic to the cell surface and to regulate γδ IEL, albeit suboptimally, leaves open the possibility that non-heteromeric complexes might be active, e.g., in disease settings in which BTNL proteins might be dysregulated.

Btnl1-dependence has to date been attributed solely to Vγ7, and so an influence of Vδ chains on the Btnl response was a priori surprising. Possibly pairings with particular Vδ chains might affect the response by altering the quarternary structures of TCRs. Alternatively, Btnl1 + 4-responsive IEL may comprise qualitatively distinct cells whose responsiveness might reflect their development along a distinct pathway: indeed, Vδ6.3 expression has been associated with PLZF-expressing innate-like lymphocytes^46^. This issue will be addressed by single-cell transcriptomics. Because the murine gut epithelium expresses Btnl1, Btnl4 and Btnl6, it is also not obvious why Btnl1 + Btnl6 is the dominant selecting combination, although this might reflect expression levels (considered above), a prospect which cannot be investigated at the protein level until appropriate reagents are available.

Intriguingly, Vγ7^+^ IEL numbers did not decline over many weeks following acute *Btnl* gene locus ablation. This was surprising given that the IEL showed reduced expression of CD122, the receptor for IL-15 which is an important IEL growth factor^47,48^. Possibly, Vγ7^+^ IEL were still able to compete for IL-15 because of the reduction in receptor expression by most such cells. Alternatively, the impact of reduced IL-15R expression on IEL might become evident by assessing the cells’ replenishment in mice following infection or injury. The Btnl-dependence of sustained CD122 expression is also interesting in the light of reports that IL-15 regulates mucosal T-cell mobility within the gut, as part of immune surveillance^47,49,50^.

In this regard, an unanticipated observation was that although Vγ7^+^ IEL numbers were maintained in mice acutely depleted of *Btnl6*, the TCRδ repertoire changed toward that seen in constitutive *Btnl6*^−/−^ mice. This presumably reflects ongoing replenishment of the gut IEL the half-lives of which have been reported to range from 2 to 14 weeks^51^. We hypothesise that during a developmental window in early life, Btnl proteins are required to drive the selective differentiation and proliferation of IEL progenitors so that mature, expansive repertoires form. Thereafter, local self-renewal occurs from a mature progenitor pool, akin to that recently identified for memory CD8 T cells^52^, that does not require sustained Btnl expression but that is nonetheless influenced by it. Hence, somatic changes in Btnl expression patterns have the potential to change the IEL repertoire and status, as occurred in this study.

This scenario may model human disease settings where BTNL protein becomes altered, e.g. by inflammation or other gut pathophysiology^24^. However, the consequences may be greater than in mouse, because to date the potential to make only one type of heteromer (BTNL3 + 8) has been identified in the human colon. Hence, the reduced expression of either BTNL protein, as has been reported in colon cancer (www.oncomine.org), might undermine the capacity to sustain the normal IEL repertoire and its functions in tissue maintenance, that have seemingly been conserved from agnathans to *Homo sapiens*. Finally, we note that future studies should investigate whether Skint/Btnl/BTNL heteromers exert cell-autonomous effects on the epithelial cells that express them, outside of the impacts on their local lymphocyte compartments.

## Methods

### RNAscope

Rnascope was performed using probes for Btnl1 and Btnl6 according to the manufacturer’s instructions. RNAscope was performed on paraffin embedded sections using probes and kits obtained from Advanced Cell Diagnostics/biotechne using the RNAscope 2.0 HD Reagent Kit-BROWN. Reference sequences are as follows: *Btnl1*, GenBank:NM_001111094.1 (576-1723); *Btnl4*, GenBank:NM_030746.1 (560-968); *Btnl6*, GenBank:NM_030747.1 (245-1552) and images were acquired using a Zeiss Axio/scan Z1 slide scanner and Zen Image acquisition software (Zen Blue, v2.6 Carl Zeiss Microscopy).

### Tissue-specific deletion of genes

Tissue-specific deletion of genes was achieved by crossing floxed Cre-transgenic lines: Pgk-Cre (MGI: 2178050), Villin-Cre (MGI: 3053819) and VillinCre/ERT2 (MGI: 3053826). Tamoxifen (Sigma, T5648) dissolved in corn oil (Sigma, C8667) was administered on consecutive days as indicated via i.p. injection and animals were sacrificed on indicated timepoints. Successful deletion was confirmed by qPCR.

### Spleen immunophenotyping

Comprehensive immunophenotyping of Btn6^−/−^ mice was performed using a platform developed by the Wellcome Trust Infection and ImmunityImmunophenotyping (3i) consortium (www.immunophenotyping.org)^53^. In brief, Spleen and MLN were digested with collagenase (1 mg/ml)/DNAse (0.1 mg/ml) in 2% FCS PBS (+Ca/Mg) for 20 min at 37 °C and filtered through 30 μm cell strainers. Cells were plated on 96-well V-bottom plates, washed in PBS and stained with Zombie Near-IR (Biolegend) for live/dead discrimination. Antibody stains were performed at 4 °C for 20 min. Full details regarding phenotyping panels are included in Table S1. Samples were acquired on a BD LSR Fortessa X-20 equipped with 405 nm (40 mW), 488 nm (50 mW), 561 nm (50 mW) and 640 nm (100 mW) lasers.

### Mice

Wild-type (WT) C57BL/6J and FVB mice were obtained from Jackson Laboratories. NF-Skint1^Tg^, Btnl1-KOMP, Btnl4-KOMP and Skint1^Δ/Δ^ mice have been previously described^16,29,32^. Genetically engineered mice were generated at the Francis Crick Institute’s Transgenic Facility. The sg RNAs & PAM sequences (see Table 1) were cloned into the g-RNA basic vector, translated in vitro, purified and co-injected with Cas9 into day 1 zygotes and transferred into pseudopregnant foster mice by the Francis Crick Institute’s Transgenic Facility. Targeted animals were identified and validated by PCR and later genotyped using the Transnetyx platform. All animals were maintained at The Francis Crick Institute’s Biological resource facilities with a 12 h light/dark cycle and access to food and water ad libitum, temperature 19–23 °C, 55 ± 10% humidity. Animal experiments were undertaken in full compliance with UK Home Office regulations and under a project license to A.C.H. (7009056).

**Table 1:** Oligos and repair templates used for generation of floxed mice.

Short guide oligo (sg) and homology repair (HDR)	Sequence 5′–3′
Btnl6-5′sg-2	TAACCTGGGGAGGAGTTAAG**AGG**
Btnl6-3′sg-2	AGGATTCACACTGACAACTT**AGG**
Btnl6-5′ HDR template	AGCAGAGATGGCTTGCGGTGATTTTCCATGTCCAGCAGAACTGAAGAGAAAAACAGGAGAGGCAGATCAATAACCTGGGTACC*ATAACTTCGTATAGCATACATTATACGAAGTTAT*GGGAGGAGTTAAGAG**A**CCAAATCCACCCAGATCTTGGACCCCTCCTCAGAGACAGCATTGC
Btnl6-3′HDR template	AGGCTCCAGGCCCTTCCAGGACCCATGGGGGCTTTGGCCTGTGGCTTCTACACTACTACAAGGATTCACACTGACAAGGTACC*ATAACTTCGTATAGCATACATTATACGAAGTTAT*CTTAGGCAGTGGTCCAGACTATGGGAACAGAGAGTTCCTGGCATGCTGGAGGAATGGAGAGTCTTC
Btnl1 Int12 sg-3	CCCAAGGGGGATCTTGGAGC**TGG**
Btnl1 Int56 sg-2	TCCATAGCACCTTATCCGGT**TGG**
Btnl1-HDR template_1	AATGTGGGAGTGGTCTACTTTCTTGTATGACTTCACTGCCCTACATTGGACTCAGAGAACCCAGCTTAATTAA*ATAACTTCGTATAGCATACATTATACGAAGTTAT*CCAAGATCCCCCTTGGGACCATGAACTCACAGAAAGGCGAGAGAAAATGGGAACTTGGCAGCTTTCCATGTCCACGG
Btnl1-HDR template_2	AAGCCCTAAGACACCTTAAACTCCCAAGGTGCTGGGACATTGCTCTGTGACTCCATAGCACCTTATTAATTAA*ATAACTTCGTATAGCATACATTATACGAAGTTAT*CCGGTTGGTGTCCCTGTGAGCATGCTCATCTCCTTTATCATGGGGCCTCTACGGGAACGCCAAGTCTAATTCGTTAG

Bold letters indicate the PAM, underlined letters indicate the restriction site and italic letters indicate loxP.

### Generation of Skint1, Skint2 rat monoclonal antibodies

Rat monoclonal antibodies against Skint1 and Skint2 were generated by immunization of Lou/c rats with purified GST-tagged human Skint1 or Skint2 extracellular domain, respectively. Hybridoma cells were generated and binding to Skint1 or Skint2 protein was analysed by enzyme-linked immunosorbent assay (ELISA). Positive hybridoma supernatants were further assayed for their potential in immunoblotting. Hybridoma clones Skint1 2G2 and Skint2 3G8 (both IgG2a/k) were recloned by limiting dilution to obtain stable monoclonal cell lines.

### Quantitative RT-PCR

Samples were stored in RNAlater (Ambion) or directly frozen in RLT buffer prior to RNA purification with DNAse digest (QIAGEN RNeasy kit). cDNA was generated using Superscript-II (Invitrogen) and analysed using Sybr-green assay (Invitrogen) using a Quant-studio 5 or Viaa7 Real-time PCR machine (Applied Biosystems) and qPCR primers indicated in Table 2.

**Table 2:** qPCR primers.

Target	Forward	Reverse
Mu-Btnl-1	TGACCAGGAGAAATCGAAGG	CACCGAGCAGGACCAATAGT
Mu-Btnl-4	CATTCTCCTCAGAGACCCACACTA	GAGAGGCCTGAGGGAAGAA
Mu-Btnl-6	GCACCTCTCTGGTGAAGGAG	ACCGTCTTCTGGACCTTTGA
Mu-Ppia	CAAATGCTGGACCAAACACAA	CCATCCAGCCATTCAGTCTTG
Mu-Skint1	AAACAAAAGGGAGCTGACCC	CCCCTCTAAGCCGTTCACTA
Mu-Skint2	GCTACAGGAGTACTTCTCTGTGTTGT	TGGTGCCAAGACTGGCCT
Mu-Psmb9	GTCGTGGTGGGCTCTGATT	GAACCTGAGAGGGCACAGAA
Mu-Btnl2	TTTGCTATGGATGACCCTGC	TCCTGATTGCTGCTGTGTGT

### Isolation of mouse intestinal IEL

Mouse IEL were isolated from small intestine^18^. Briefly, small intestine was opened, washed in PBS, cut into 0.5-cm long pieces and incubated at RT on awheel in complete RPMI supplemented with 1 mM DTT. Tissues were then washed, vortexed in complete RPMI and filtered through 70 nm nylon cell strainers. Vortexing and filtration steps were repeated twice. IEL were then purified by Percoll density centrifugation and stained by flow cytometry (for antibodies, see Table 3).

**Table 3:** Antibodies used for flow cytometry, western blotting and microscopy.

Antibodies	Clone	Source	Identifier/Cat no.	Dilution
CD3 APC Cy7	17A2	BioLegend	100222	1:400
TCRβ BV421	H57-597	BioLegend	109229	1:300
CD122 PE	TM-β1	BioLegend	123209	1:200
Thy1.2 BV 510	53-2.1	BioLegend	140319	1:800
Lag3PerCPefluor 710	C9B7W	eBioscience	46-2231-80	1:300
CD24-BV650	M1/69	BD	563545	1:400
CD8α PECy7	53-6.7	BioLegend	100722	1:200
TCR Vδ4 FITC	GL-2	BD	552143	1:100
TCR Vδ4 PE	GL-2	BioLegend	134905	1:100
CD8β PerCpCy5.5	YTS156.7.7	BioLegend	126610	1:200
TCR Vγ1.1/Cr4 FITC	2.11	BioLegend	141103	1:100
TCR Vγ4 APC	UC3-10A6	BioLegend	137708	1:100
TCRδ BV421	GL3	BioLegend	118119	1:200
TCRδ Pe	GL3	BioLegend	118108	1:800
TCRδ PeCy7	GL3	BioLegend	118124	1:200
CD4 BV 510	RM4-5	BioLegend	100559	1:100
TCR Vδ6.3/2 BV711	8F4H7B7	BD	744476	1:100
CD25-PerCP/Cy5.5	PC61	BioLegend	102030	1:200
TCR Vδ6.3/2-PE	8F4H7B7	Pharmingen	555321	1:300
TCR Vγ7	F2.67	Institut Pasteur, Paris, P. Pereira	N/A	1:400
CD45Rb-FITC	C363.16 A	eBioscience	11-0455-82	1:100
Vγ5-APC	7-17	BioLegend	137506	1:100
TCRδ PerCPeFluor710	GL3	eBioscience	46-5711-82	1:200
TCRδ AF647	GL3	BioLegend	118134	1:200
CD45 PB	HI30	BioLegend	304022	1:300
CD69 PE	H1.2f3	eBioscience	12-0691-93	1:200
CD3 PerCPCy5.5	SK7	BioLegend	344808	1:300
DYDDDDK PeCy7	L5	BioLegend	637324	1:300
HA AF647	16B12	BioLegend	682404	1:200
HIS PE	J095G46	BioLegend	362603	1:100
CD24-BV650	M1/69	BD	563545	1:400
CD62L-BV421	MEL-14	BioLegend	104436	1:300
CD44-Pe-Cy7	IM-7	BioLegend	103030	1:300
CD45 eVolve 605	30-F11	eBioscience	83-0451-42	1:100
TCR Vγ5-PE	536	BioLegend	137504	1:100
Vγ5Vδ1	17D1, Supernatant	Yale, US, R. Tigelaar, J. Lewis	N/A	1:2
TCR-Vg5-FITC	536	BD	553229	1:300
MHC I-A/I-E-AF647	M5/114.15.2	BioLegend	107618	1:500
CD45-eFluor 450	30-F11	eBioscience	48-0451-82	1:200
Skint1	2G2	Monoclonal Antibody core facility (Helmholtz Zentrum Munich)		100ul SN /IP
Skint2	3G8	Monoclonal Antibody core facility (Helmholtz Zentrum Munich)		1:1000
Flag	M2	Merck	F1804	1:5000
Goat anti rat HRP		Thermo Fisher	31470	1:5000
Goat anti mouse HRP		Thermo Fisher	31446	1:5000
Flag magnetic beads	M2	Sigma	M8823	25 µl/IP

#### IEL cultures

IEL cultures were performed^19^. Briefly, 10^5^ MODE-K cells were plated onto 48-well plates 24 h prior to co-culture experiments. The following day, the medium was removed and 10^5^ unsorted IEL suspended in 200 µl of RPMI 1640 supplemented with L-glutamine, 10% heat-inactivated FCS, 1% pen/strep, 10 mM hepes, 1 mM sodium pyruvate, 1x non-essential amino acids, 50 µM β-mercaptoethanol, IL-2 10 U/ml, IL-15 10 ng/ml (Immunotools), IL-3 100 U/ml and IL-4 200 U/ml (R&D) were seeded on top of the monolayer. Cells were co-cultured overnight (16–18 h) at 37 °C with 10% CO_2_.

Flow Cytometry acquisition was performed using BD-FACS/Diva Software. Data analysis was performed using FlowJo v10 10.6.1 (FlowJo, LLC, Ashland OR).

### Cell lines

HEK293T cells (FCI) were maintained in DMEM supplemented with 4.5 g/l d-glucose, l-glutamine, 10% heat-inactivated FCS and 1% penicillin-streptomycin (complete DMEM). Transgenic MODE-K cell lines^19^ were maintained in complete DMEM supplemented with 1 μg/ml puromycin (Sigma-Aldrich) and 500 μg/ml hygromycin (Thermo Fisher). Transgenic J76 cells^19^ were maintained in RPMI 1640 l-glutamine, 10% FCS and 1% penicillin-streptomycin. All cell culture reagents were from Thermo Fisher.

#### Cell line co-culture

In all, 5 × 10^4^ transduced J76 was mixed in 96-well plates with 2 × 10^5^ transiently transfected 293T cells, followed by co-culture for 5 h.

### Plasmids and transfection

Overlap-extension PCR (OE-PCR) was used to replace the GFG regions of Btnl4 with those of Btnl1 on plasmids encoding Btnl1/4/6^16^. HEK293T cells were transfected with the indicated combinations of FLAG-Btnl1, HA-Btnl6, HIS-Btnl4 and empty vector (EV) encoding plasmids. Medium was replaced 16 h after transfection and cells were harvested at 48 h and used for the co-culture assay. For antibodies see Table 3.

### Preparation of epidermal sheets

Ear epidermis was separated from dermis following incubation in 0.5 M ammonium thiocyanate for 35 min at 37 °C. Isolated epidermal sheets were fixed with ice cold acetone at −20 °C. The samples were blocked in 5% FCS for 1 h at room temperature and stained for 1 h at 37 °C using Vγ 3 TCR-FITC (clone 536, BD), MHC I-A/I-E-AF647 (clone M5/114.15.2, BioLegend) and CD45-eFluor450 (clone 30-F11, eBioscience) antibodies. Tissue samples were mounted on microscope glass in Prolong Gold mounting medium under a stereomicroscope to ensure flat epidermal mounting. Confocal images were recorded using Leica SP5 confocal microscope with 40× 1.25 NA HCX PL APO CS lens. Three confocal records 387.5 × 387.5 µm size were acquired from each epidermal sheet. Image quantification was performed using Definiens Developer software (version XD2.7). Each channel in a record was processed with Gaussian filter followed by application of automated multi-threshold segmentation. Individual cells (CD45+, Langerhans cells, and T cells) were detected based on their relative intensity in CD45, MHC II and TCR channels, respectively. Cell number and morphology were measured for each cell type.

### Preparation of lung and uterus γδ cells

Lungs and uteri from experimental mice were collected in medium and minced with razor blades. Samples were digested using Miltenyi Multi Tissue Dissociator kit 1, according to the manufacturer’s instructions. Briefly, samples were transferred to GentleMACS C tubes containing 2.5 mL digestion mix (100 µL Enzyme D, 50 µL Enzyme R and 12.5 µL Enzyme A) and incubated at 37 °C for 40 min with shaking. Following incubation, single-cell suspensions were prepared by homogenisation using GentleMACS program C and filtering through 70 µm cell strainers. Single-cell suspensions were stained with Live/Dead Aqua for dead cell exclusion, followed by Fc-block and surface stain with specific antibodies.

### Biochemistry

Cells were lysed for 30 min in ice cold RIPA buffer with protease inhibitors (Roche) and phosphatase inhibitors (Phosphatase inhibitor cocktails 2 & 3, Sigma) and spun at 20,000 × *g* for 15 min at 4 °C. Protein concentrations in supernatants was determined using a BCA kit (Pierce).

#### Immunoprecipitation from cell lysates

Lysates were precleared on Protein G Sepharose (Millipore-Sigma) for 1 h, incubated with antibodies for 1 h followed by incubation with 1% BSA blocked Protein G beads for a further hour. Following three washes in RIPA buffer immunoprecipates or samples were mixed with NuPAGE LDS Sample Buffer supplemented with 1x NuPAGE reducing agent and separated by electrophoresis on NuPage 4–12% Bis-Tris protein gels (Thermo Fisher) and then transferred onto PVDF membranes. Membranes were blocked in PBS-0.1% Tween20 and 5% BSA for 60 min at room temperature and incubated with primary antibodies overnight. Membranes were washed three times with PBS-0.1% Tween20 and incubated for 60 min with HRP conjugated secondary antibodies, washed again and developed using ECL detection reagents (Merck).

#### Immunoprecipitation from thymus

12 Thymi of E17/E18 FVB and 22 Thymi of NF-Skint1^Tg^ pups were lysed as described above and incubated with Flag-M2 coated beads overnight at 4 °C. Following three washes in RIPA buffer immunoprecipates were eluted with 3xFlag peptide to obtain the eluate or beads were directly boiled for analysis. Beads, Eluate or samples were mixed with NuPAGE LDS Sample Buffer supplemented with 1x NuPAGE reducing agent and separated by electrophoresis on NuPage 4–12% Bis-Tris protein gels (Thermo Fisher) and then transferred onto PVDF membranes. Membranes were blocked in PBS-0.1% Tween20 and 5% BSA for 60 min at room temperature and incubated with primary antibodies overnight. Membranes were washed three times with PBS-0.1% Tween20 and incubated for 60 min with HRP conjugated secondary antibodies, washed again and developed using ECL detection reagents (Merck).

### Bioinformatics analysis

Raw gene counts were obtained from GSE109413 (Moor et al.)^54^ and GSE92332 (Haber et al.)^55^ and each cell-set was preprocessed using Seurat^56^ (version 3.1.1.9023). In the case of data from Moor et al. data, cells with <200 and >3000 detectable genes and cells with percentage mitochondrial expression greater 5% were removed. For all cell-sets, the total counts were scaled to 1e4 counts, a log transformation applied and genes were z-score across all cells. In the case of data from Moor et al., both replicate cell-sets were merged using Seurat’s IntegrateData function after which the gene-wise vectors were rescaled. The dot plots were produced using the DotPlot function from Seurat and profile plots were produced across the villus regions using the z-score scaled data.

### Statistical analysis

Summary data are represented as mean ± SD if representative experiments are shown or mean ± SEM if summarized data are shown as indicated in individual figures. Numbers of animals per group are indicated in individual figures.

Control groups includes animals that are wt, heterozygous or homozygous without the respective Cre transgene. Heterozygous animals are comparable to WT animals.

### Modelling software

Figures for all modelling data were generated in PyMOL v2.0.7 (Schrodinger LLC). 3D-JIGSAW was used to generate 3D models of proteins and perform docking simulations, respectively.

### Reporting summary

Further information on research design is available in the Nature Research Reporting Summary linked to this article.

## Supplementary information

Supplementary Information

Reporting Summary

## Data Availability

This work did not include any data which mandated deposition in public databases. Associated raw data are provided in the main and/or supplementary figures. Relations to summary data charts are indicated and a full list of figures with associated raw data is provided in the reporting summary linked to this article. Raw gene counts were obtained from GSE109413 (Moor et al.)^54^ and GSE92332 (Haber et al.)^55^. For bioinformatics single-cell analysis scripts are available on github: https://github.com/ajandke/Jandke_etal_naturecomms. Immunophenotyping data for pipeline procedure can be found under https://www.mousephenotype.org/data/secondaryproject/3i.

## Code availability

The full source codes have not been released. Publicly available servers can be accessed online for 3D-JIGSAW (https://bmm.crick.ac.uk/~svc-bmm-3djigsaw/). For bioinformatics single-cell analysis, scripts are available on github: https://github.com/ajandke/Jandke_etal_naturecomms.
