# Supplementary material for: Butyrophilin-like proteins display combinatorial diversity in selecting and maintaining signature intraepithelial γδ T cell compartments
Source: Nat Commun. 2020 Jul 28;11:3769. doi: 10.1038/s41467-020-17557-y (PMC7387338; doi:10.1038/s41467-020-17557-y)
Supplement: Supplementary file 1 — Supplementary Information [file 41467_2020_17557_MOESM1_ESM.pdf]

Supplementary Information for:

Butyrophilin-like proteins display combinatorial diversity in selecting and maintaining signature intraepithelial $\gamma\delta$ T cell compartments

Jandke et al

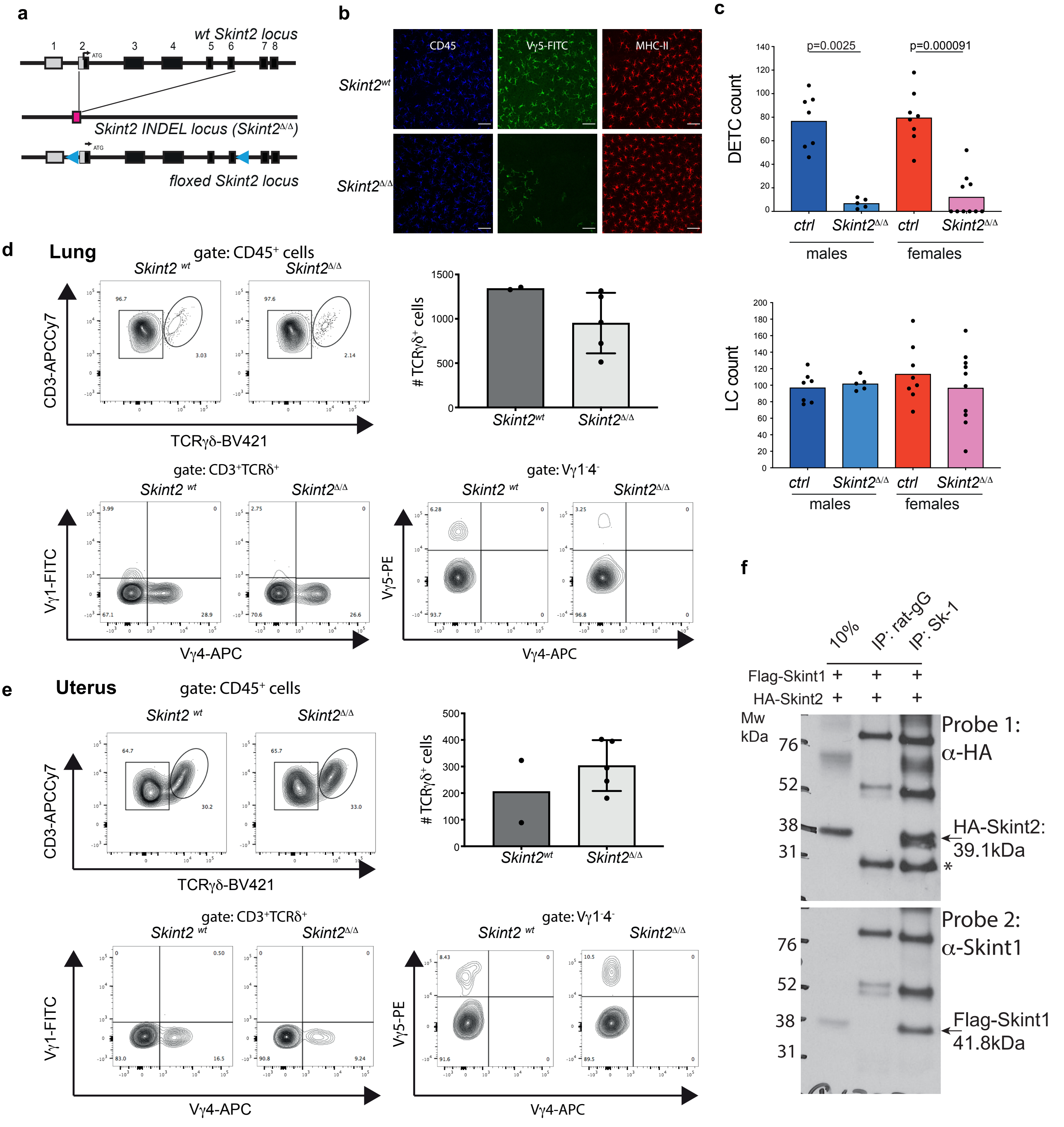

Supplementary Figure 1: Uterine and lung γδ T cells are normal in Skint2$^{\Delta/\Delta}$ animals

a Targeting strategy to generate Skint2-deleted and Skint2-floxed mice. Black: translated exons, grey: untranslated regions, blue triangles: loxP sites, red rectangle: indel region. **b** Microscopy images of adult mouse ear epidermal sheets in control and *Skint2*$^{\Delta/\Delta}$ mice. Comparison of DETC stained for CD45 (left: blue) and Vγ5$^+$ (middle: green) MHC-II$^+$) cells (bottom:red).scale bar: 50μm **c** Quantification of DETC and Langerhans cell counts split by gender of samples represented in Fig 1F, n_{wt},male:7,n_{ko} males: 5, n_{wt} females:8,n_{ko} females:10, Mann-Whitney analysis, two sided. Data are from 2 (ctrl females, indel/indel males) or 3 (ctrl males, indel/indel females) independent experiments **d** Analysis and quantification of lung γδ cells in wt and *Skint2*$^{\Delta/\Delta}$ animals. No statistical significant difference was detected n_{wt}:2, $n_{Skint2\Delta/\Delta}$:5, (unpaired two tailed t-test). Representative data from 2 experiments. **e** Analysis and quantification of uterine γδ cells in wt and *Skint2*$^{\Delta/\Delta}$ animals. No statistical significant difference was detected, n_{wt}:2, $n_{Skint2\Delta/\Delta}$:5), (unpaired two tailed t-test). Representative data from 2 experiments. **f** Immunoprecipitation of Flag-Skint1 with anti-Skint1 antibody. 293T cells which were transiently cotransfected with Flag-Skint1 and HA-Skint2 were subjected to immunoprecipitation with α-Skint1 antibody and probed for Flag and Skint1 to detected the tagged proteins. Left lane: Input , Middle lane: Rat IgG isotype control, Right lane: Skint1-IP. Askterisk: IgG-light chain, representative blot of 3 independent experiments.

Supplementary Figure 1

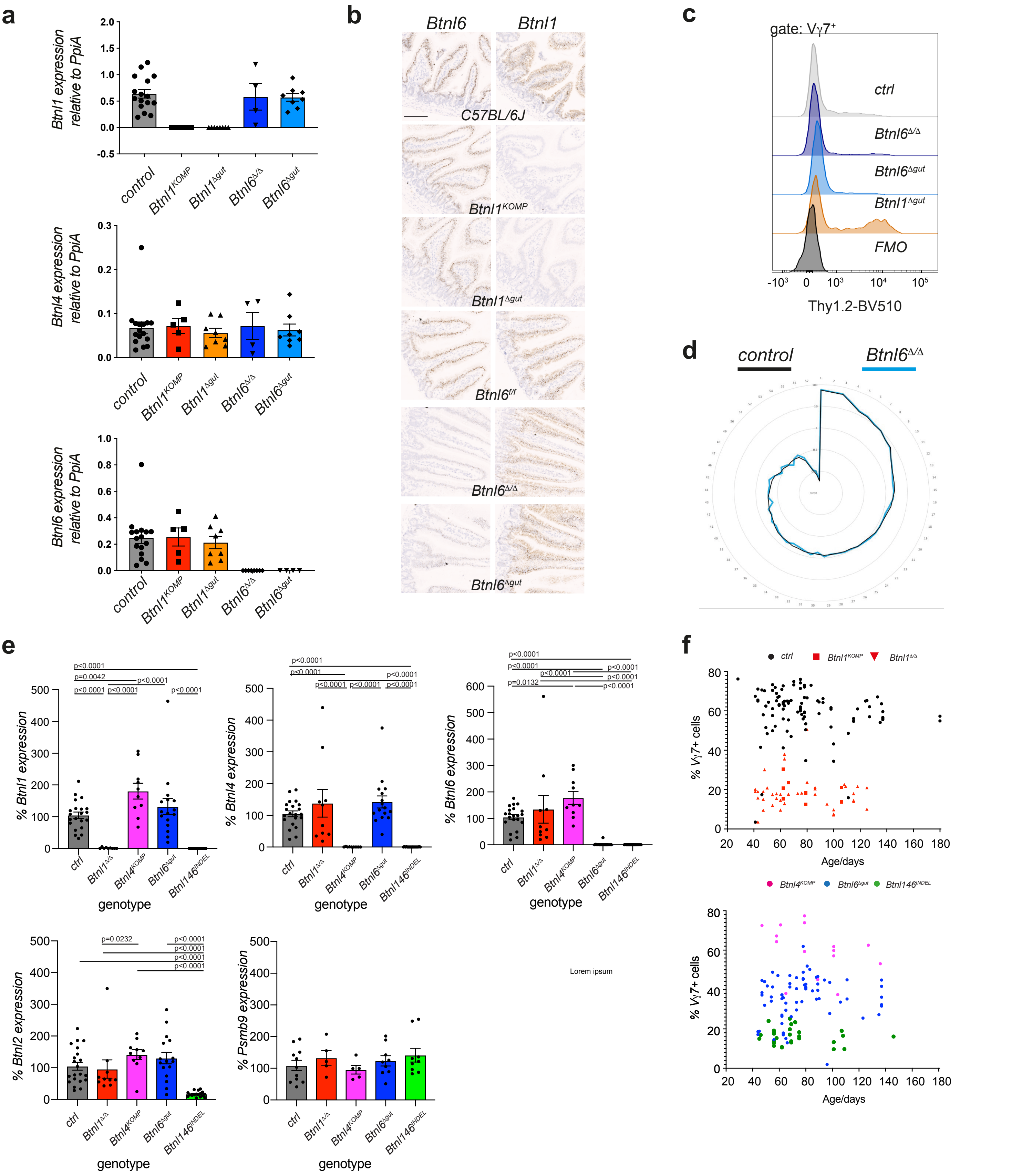

Supplementary Figure 2: Characterisation of Btnl knockout mice

a Quantification of Btnl1(top) Btnl4(middle) and Btnl6(bottom) message levels in animals of indicated genotypes by qPCR and normalised to Ppia. Data are Mean +/- SEM of 3 independent experiment. n_{ctrl}:16, $n_{Btnl1KOMP}$:5, $n_{Btnl1\Delta gut}$:8, $n_{Btnl6\Delta/\Delta}$:4, $n_{Btnl6\Delta gut}$:8. **b** RNAscope images to detect message for Btnl1 (right) or Btnl6 (left) on gut sections from animals of indicated genotypes (sections stained/genotype n_{ctrl}:3, n:$_{Btnl1KOMP}$:3,$n_{Btnl1\Delta gut}$:2, $n_{Btnl6\Delta/\Delta}$:2, $n_{Btnl6\Delta gut}$:2,1 experiment). scale bar 100µm **c**Thy1.2 surface phenotype in TCRβ$^-$CD3$^+$Vγ7$^+$ cells in animals of indicated genotypes. **d** Comparison of lymphocyte populations wt and *Btnl6*$^{\Delta/\Delta}$ animals using the previously described phenotyping panel from the 3i consortium mouse screen[1]. For individual annotation of populations see Supplementary table 1. For each group: n=7. **e** qPCR analysis of Btnl gene expression in animals of indicated genotypes normalised to mean values of control cohorts. Data are Mean +/- SEM of 3 (Psmb9) or 5 (all other genes) independent experiment. Btnl1/4/6/2 qPCRs: n_{ctrl}:21, $n_{Btnl1\Delta/\Delta}$:10, , n:$_{Btnl4KOMP}$:10, $n_{Btnl6\Delta/\Delta}$:16, $n_{Btnl146Indel}$:17. Psmb9 qPCR: n_{ctrl}:11, nBtnl1$_{Btnl1\Delta/\Delta}$:5, , $n_{Btnl4KOMP}$:5, $n_{Btnl6\Delta/\Delta}$:9 $n_{Btnl146Indel}$:9. Statistical analysis: two-tailed Mann Whitney test. **f** Comparison of %Vγ7$^+$ cells in animals of indicated genotypes over age. n_{ctrl}:98, $n_{Btnl1KOMP}$:9, $n_{Btnl1\Delta/\Delta}$:41, $n_{Btnl4KOMP}$:15, $n_{Btnl6\Delta/\Delta}$:68 $n_{Btnl146Indel}$:30.

Supplementary Figure 2

a

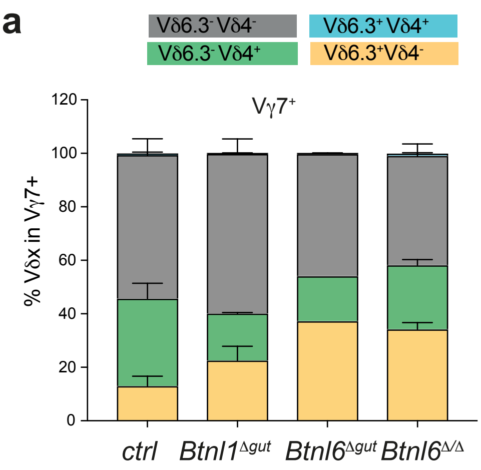

compared groups	$V\delta6.3^+V\delta4^-$	$V\delta6.3^+V\delta4^+$	$V\delta4^+V\delta6.3^-$	$V\delta6.3^-V\delta4^-$
ctrl *Btnl1*$^{\Delta gut}$	p=0.0102	ns	p=0.000018	ns
ctrl *Btnl6*$^{\Delta gut}$	p<0.00001	ns	p=0.000146	ns
ctrl *Btnl6*$^{\Delta/\Delta}$	p<0.00001	ns	p=0.02194	p=0.000335
Btnl1$^{\Delta gut}$ *Btnl6*$^{\Delta gut}$	p=0.00456	ns	ns	p=0.007436
Btnl1$^{\Delta gut}$ *Btnl6*$^{\Delta/\Delta}$	p=0.01485	ns	ns	p=0.000027
Btnl6$^{\Delta gut}$ *Btnl6*$^{\Delta/\Delta}$	ns	ns	ns	ns

c

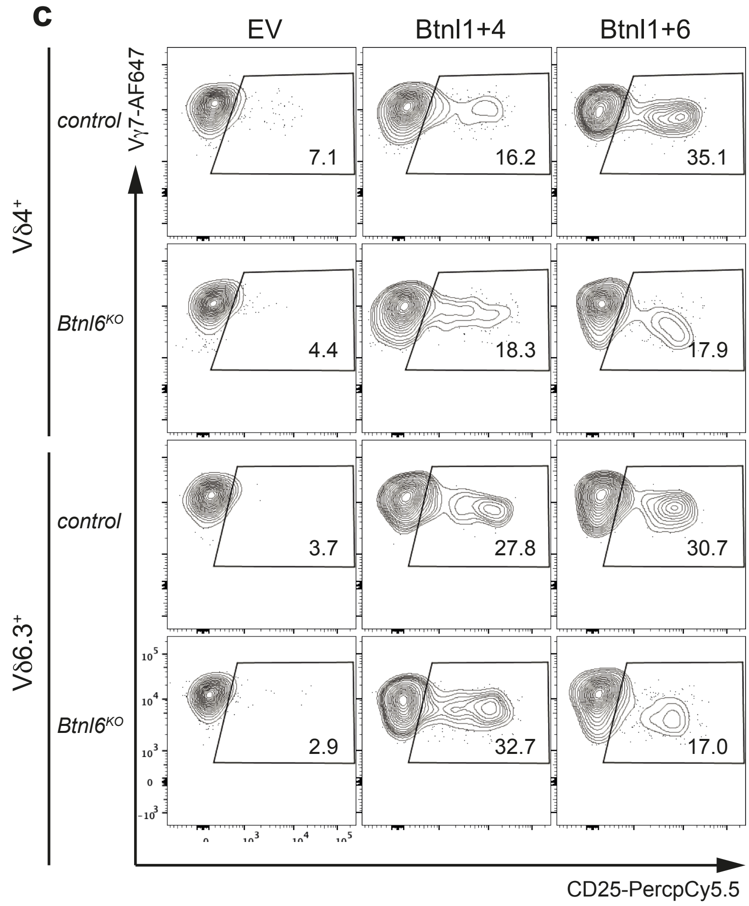

b

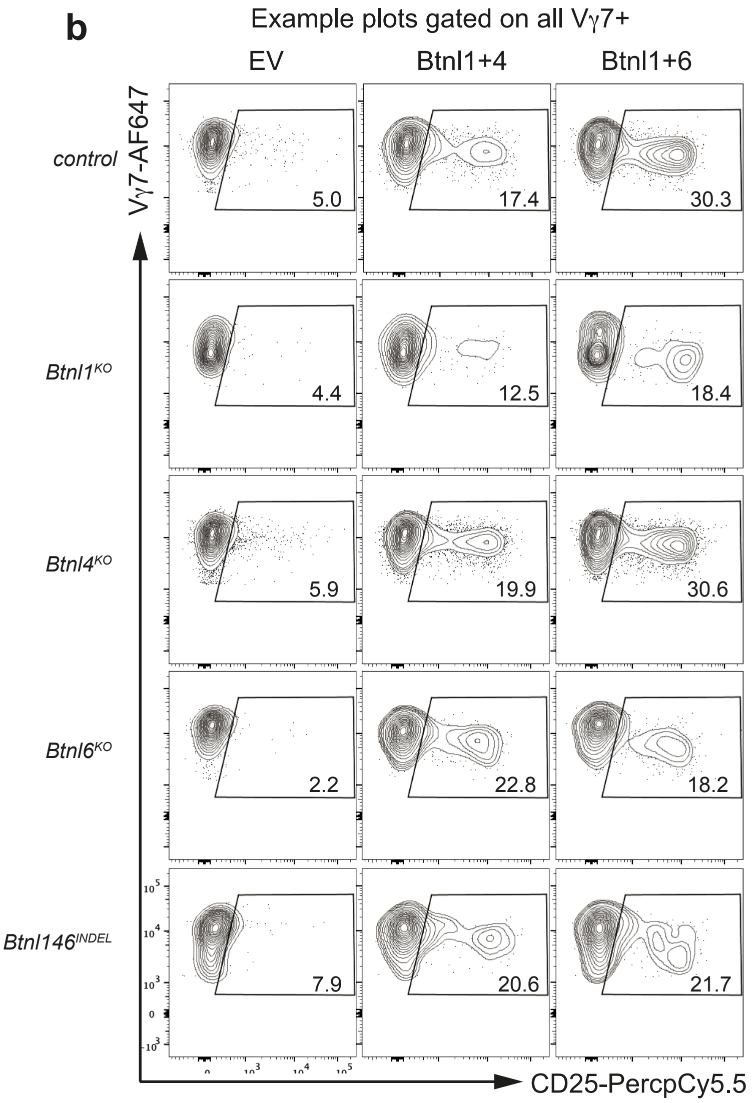

d

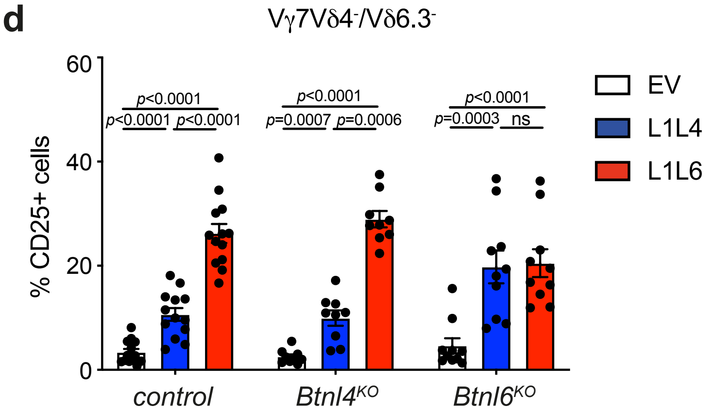

Supplementary Fig 3: γδ usage in Btnl6 deficient strains and example flow cytometry plots for data summarized in Figure 4

a Quantification of Vδ-chain usage in $V\gamma7^+$ IEL as depicted in quadrants in Fig3a. n_{ctrl}:12, n_{Btnl1}:3, $n_{Btnl6\Delta gut}$:2, $n_{Btnl6\Delta/\Delta}$:3. Statistical analysis 2-way ANOVA & Tukey's multiple comparison post test. **b** Example Flow cytometry plots corresponding to quantification graphs in Fig 4D. Numbers in gates indicate % of $CD25^+$ cells. **c** Example Flow cytometry plots for coculture experiments with IEL from wild-type and Btnl6 knockout animals in indicated populations corresponding to quantification graphs in Fig 4E. top two rows: $V\gamma7^+V\delta4^+$, bottom two rows: $V\gamma7^+V\delta6.3^+$. Numbers in gates indicate % of CD25+ cells. **d** IEL response to MODE-K cells expressing different Btnl dimers (L1L4 or L1L6 or EV as control) was measured as $\%CD25^+$ cells gated on $V\gamma7^+V\delta4^-V\delta6.3^-$ cells in animals of indicated genotypes. Data are Mean +/- SEM of 5 independent experiments, n_{ctrl}:13, $n_{Btnl4KO}$:9, $n_{Btnl6KO}$:10. Statistical analysis 2-way ANOVA & Tukey's multiple comparison post test.

Supplementary Figure 3

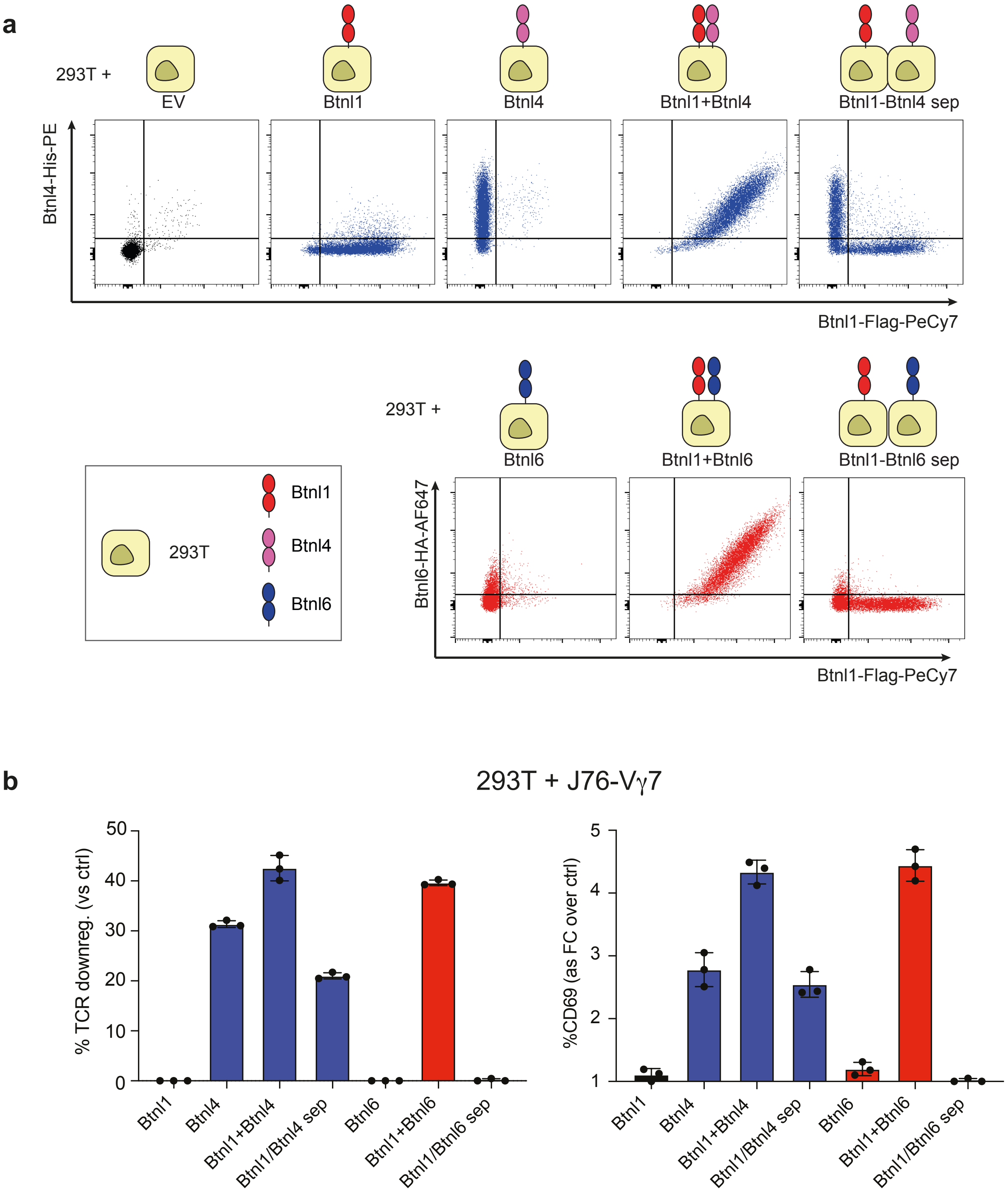

Supplementary Figure 4: Coexpression of Butyrophilins is essential for efficient TCR downregulation
a Flow Cytometry plots of 293T cells expressing either individual tagged Btnl molecules or combinations of Btnl molecules according to the scheme depicted above the Flow Cytometry plots. Coexpression of Btnls on the same cell is indicated as Btnl1+Btnl4 or Btnl1+Btnl6, expression on separate cells is indicate as sep. Top row (blue): individual and Btnl4 containing, Bottom row (red): Btnl6 containing 293T cells.
b TCR downregulation (left) and CD69 upregulation (right) by J76 cells expressing a Vγ7Vδ4 TCR and co-cultured with 293T transfected with Btnls in combinations depicted in A. Results are normalised to 293T.EV. Data are represented as Mean +/- SD of triplicate co-cultures, representative of n=3 independent experiments.

Supplementary Figure 4

a

b

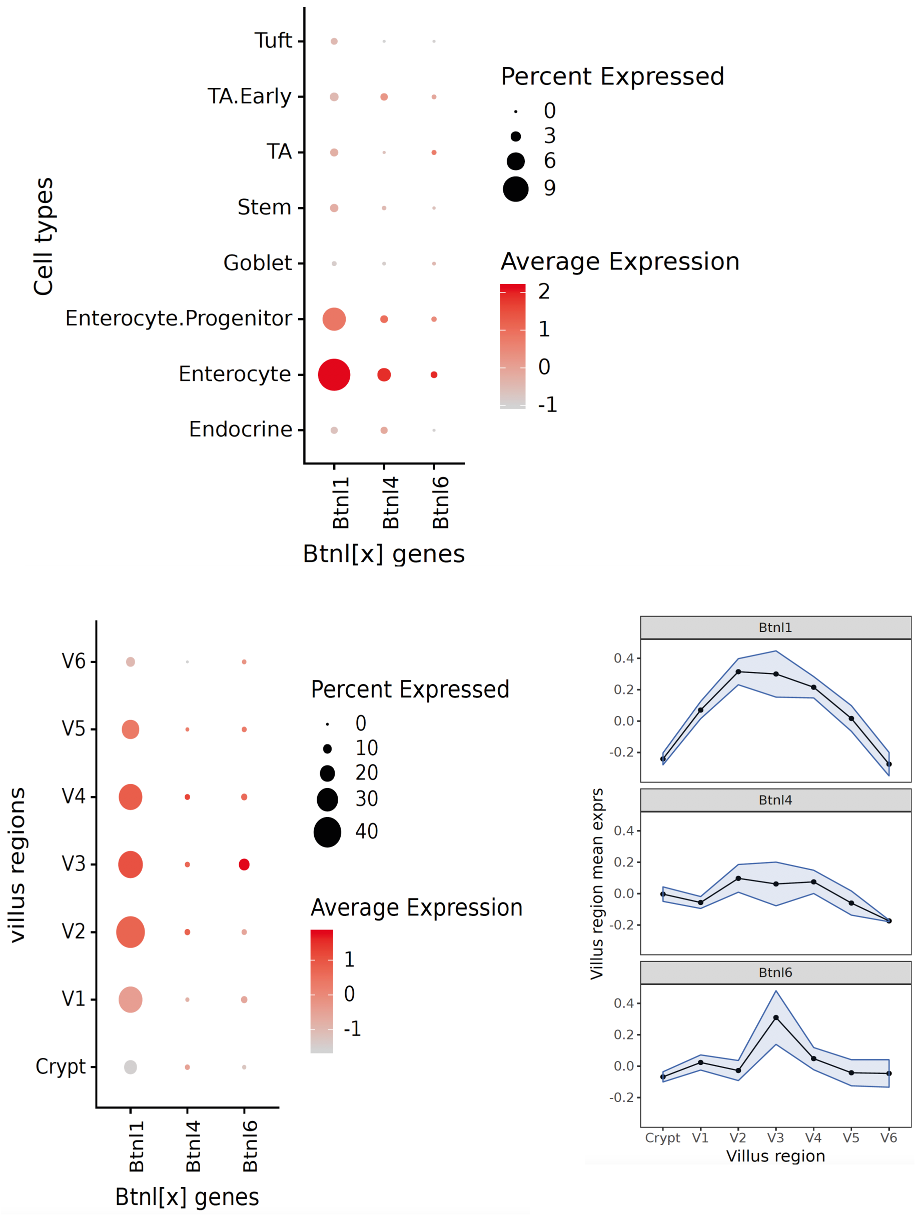

Supplementary Figure 5: Btnl expression analysis in single cell datasets
a Expression patterns of specific btnl genes in different intestinal cell population according to the dataset published by Haber et al.55 Each dot represents the indicated btnl, of which the color saturation indicates the average expression level (scaled by Z-score) and the size indicates the percentage of cells expressing the gene.
b Left: Expression patterns of specific btnl genes in different villus segments according to dataset published by Moor et al.54 Each dot represents the indicated btnl, of which the color saturation indicates the average expression level (scaled by Z-score) and the size indicates the percentage of cells expressing the gene.
Right: Mean btln z-score scaled expression across the different villus regions. The blue ribbon indicates the standard error in expression across the cells from each region.

Supplementary Figure 5

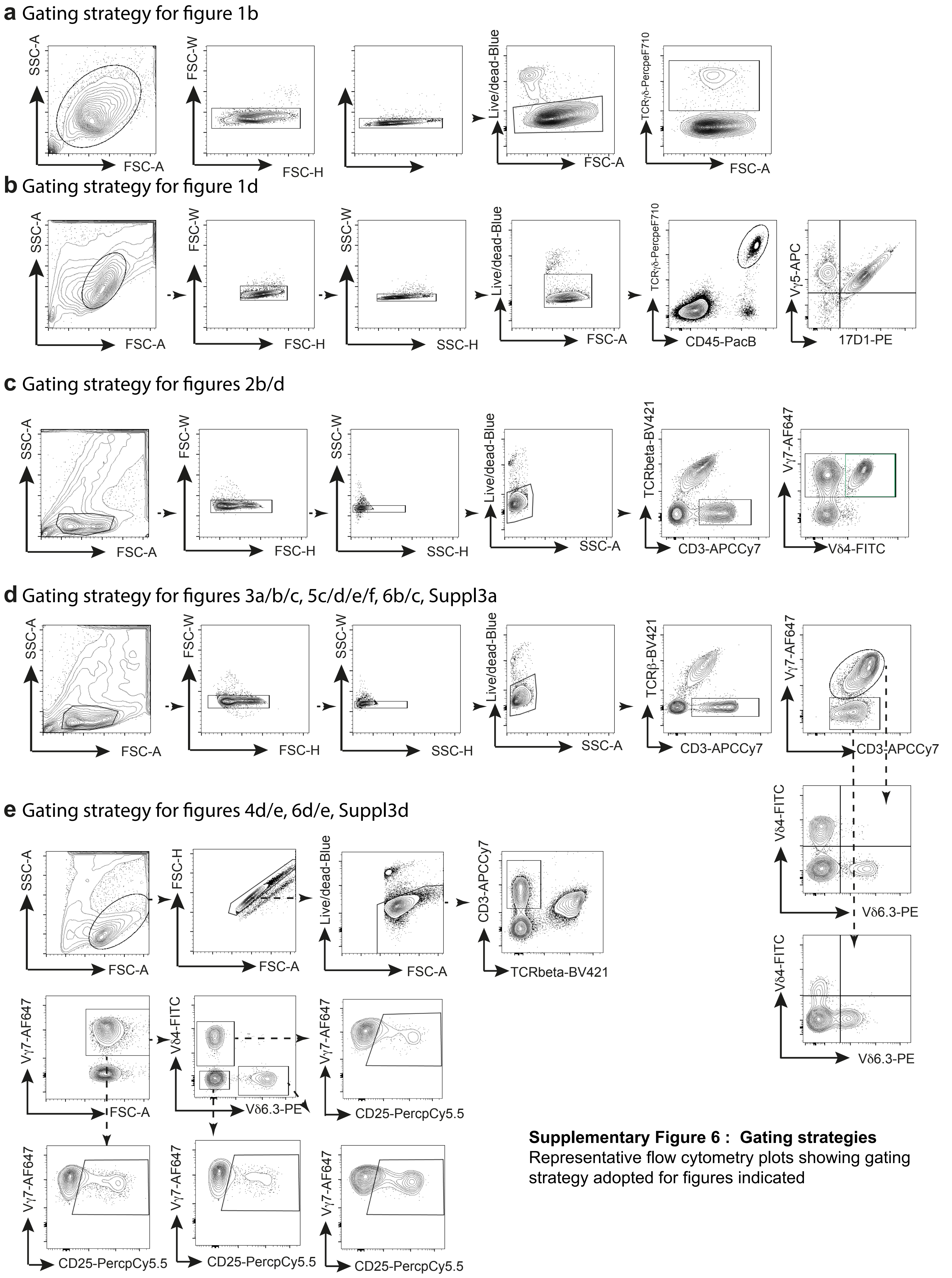

Supplementary Figure 6 : Gating strategies
Representative flow cytometry plots showing gating strategy adopted for figures indicated

Supplementary Figure 6

a

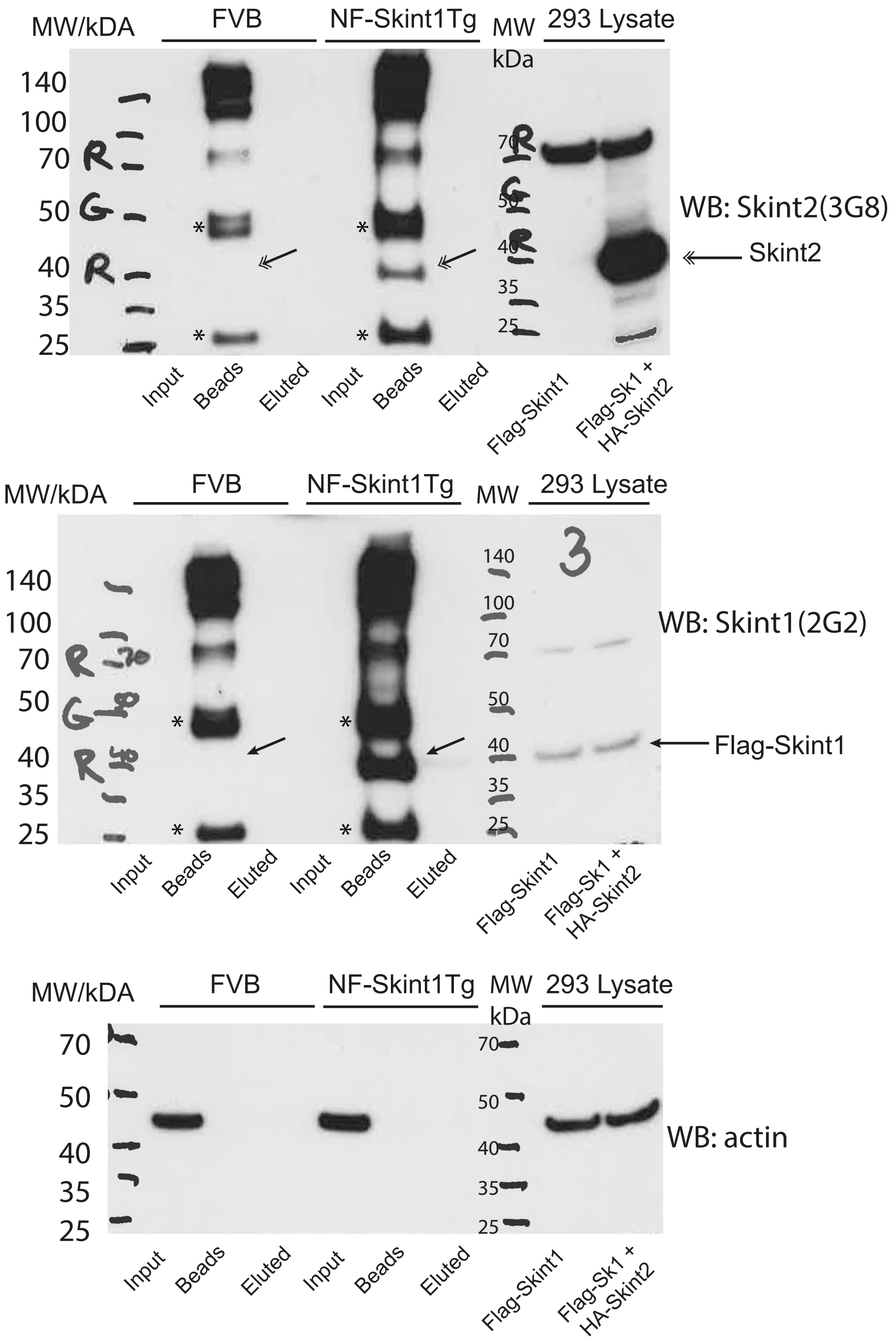

Supplementary Figure 7 : Western Blots

a) Immunoprecipitation of Flag-tagged Skint1 from FVB or NFSkint1Tg animals. Left: scheme of FVB mice expressing Skint1 and Skint2, Scheme: Top: wt FVB mice express endogenous, untagged Skint1 and Skint 2, Bottom: NFSkint1Tg animals express a Flag tagged Skint1 and untagged Skint2 on the Skint1Tac background. Right: Immunoprecipitation with anti Flag antibody from lysates of pooled thymi of FVB and NFSkint1Tg animals, (n_{FVB}=12, $n_{NFSkint1Tg}$=22, 1 experiment). Expression control in 293 lysates transduced with either Flag-Skint1 alone or Flag-Skint1 & HA-Skint2 constructs. Long arrows: Skint1 band, askterisks: non-specific bands reflecting anti-FLAG Ig chain detection.

Supplementary Figure 7

Supplementary Table 1: Cell populations analysed in Supplementary Figure 2d

Population No.	Population (% of CD45)
1	Total B2 cells
2	B2 FOI and FOII and GC B cells
3	Follicular B cells
4	Total ab T cells
5	Total $CD4^+$ T cells
6	$CD4^+$ T helper cells
7	Resting $CD4^+$ T helper cells
8	Total $CD8^+$ T cells
9	Naive $CD8^+$ T cells
10	Transitional B cells
11	Transitional 1 B cells
12	Effector $CD4^+$ T helper cells
13	Transitional 2 B cells
14	B2 marginal zone and precursor cells
15	Resting NK cells
16	Granulocytes and monocytes
17	Marginal zone B cells
18	Klrg1 NK cells
19	Granulocytes
20	Resting $CD8^+$ T cells
21	Total Tregs
22	Eosinophils
23	Total $\gamma\delta$ T cells
24	Total DC
25	Germinal centre B cells
26	Early germinal centre B cells
27	Resting Treg cells
28	Conventional DC
29	Monocytes
30	Marginal zone precursor B cells
31	Resting $\gamma\delta$ T cells
32	B1a cells
33	Effector NK cells
34	Effector CD8+ T cells
35	$CD5^+$ $\gamma\delta$ T cells
36	Effector Treg cells
37	Conventional CD11b type DC
38	Macrophages
39	Total $CD4^+$ NKT cells
40	Effector $\gamma\delta$ T cells
41	Total $CD4^-$ NKT cells
42	Plasma cells
43	Conventional $CD8\alpha$ type DC
44	Effector $CD4^+$ NKT cells
45	Resting $CD4^-$ NKT cells
46	Plasmacytoid DC
47	Memory B cells
48	$CD103^+$ $CD8\alpha$ type DC
49	Resting $CD4^+$ NKT cells
50	Effector $CD4^-$ NKT cells
51	$Klrg1^+$ $CD8^+$ T cells
52	$Klrg1^+$ Treg cells
53	$Klrg1^+$ $CD4^+$ T helper cells
54	$Klrg1^+$ $CD4^+$ NKT cells
55	$Klrg1^+$ $CD4^-$ NKT cells
56	Late germinal centre B cells
57	$Klrg1^+$ $\gamma\delta$ T cells

Supplementary References

[1-3]

1 Abeler-Dorner, L. *et al.* High-throughput phenotyping reveals expansive genetic and structural underpinnings of immune variation. *Nat Immunol* **21**, 86-100, doi:10.1038/s41590-019-0549-0 (2020).

2 Haber, A. L. *et al.* A single-cell survey of the small intestinal epithelium. *Nature* **551**, 333-339, doi:10.1038/nature24489 (2017).

3 Moor, A. E. *et al.* Spatial Reconstruction of Single Enterocytes Uncovers Broad Zonation along the Intestinal Villus Axis. *Cell* **175**, 1156-1167 e1115, doi:10.1016/j.cell.2018.08.063 (2018).
