# Supplementary material for: Butyrophilin-like proteins display combinatorial diversity in selecting and maintaining signature intraepithelial γδ T cell compartments
Source: Nat Commun. 2020 Jul 28;11:3769. doi: 10.1038/s41467-020-17557-y (PMC7387338; doi:10.1038/s41467-020-17557-y)
Supplement: Supplementary file 2 — Reporting Summary [file 41467_2020_17557_MOESM2_ESM.pdf]

nature research

Corresponding author(s): Adrian Hayday

Last updated by author(s): 2020/0616

Reporting Summary

Nature Research wishes to improve the reproducibility of the work that we publish. This form provides structure for consistency and transparency in reporting. For further information on Nature Research policies, see Authors & Referees and the Editorial Policy Checklist .

Statistics

For all statistical analyses, confirm that the following items are present in the figure legend, table legend, main text, or Methods section.

n/a	Confirmed	
☐	☒	The exact sample size (*n*) for each experimental group/condition, given as a discrete number and unit of measurement
☐	☒	A statement on whether measurements were taken from distinct samples or whether the same sample was measured repeatedly
☐	☒	The statistical test(s) used AND whether they are one- or two-sided<br>*Only common tests should be described solely by name; describe more complex techniques in the Methods section.*
☒	☐	A description of all covariates tested
☒	☐	A description of any assumptions or corrections, such as tests of normality and adjustment for multiple comparisons
☐	☒	A full description of the statistical parameters including central tendency (e.g. means) or other basic estimates (e.g. regression coefficient) AND variation (e.g. standard deviation) or associated estimates of uncertainty (e.g. confidence intervals)
☐	☒	For null hypothesis testing, the test statistic (e.g. *F*, *t*, *r*) with confidence intervals, effect sizes, degrees of freedom and *P* value noted<br>*Give P values as exact values whenever suitable.*
☒	☐	For Bayesian analysis, information on the choice of priors and Markov chain Monte Carlo settings
☒	☐	For hierarchical and complex designs, identification of the appropriate level for tests and full reporting of outcomes
☒	☐	Estimates of effect sizes (e.g. Cohen's *d*, Pearson's *r*), indicating how they were calculated

Our web collection on statistics for biologists contains articles on many of the points above.

Software and code

Policy information about availability of computer code

Data collection	Data analysis: Flow cytometry instruments were running BD FACSDiva software.<br>Pictures for RNAscope staining were acquired using a Zeiss Axio/scan.Z1 slide scanner and the Zen image acquisition and analysis software (Zen Blue, v2.6, Carl Zeiss Microscopy). quantitative PCR were run on QuantStudio5 qPCR machines (ThermoFisherScientific).
Data analysis	Molecular Modelling was performed using 3D-JIGSAW and SwarmDock (references provided). The source codes have not been released. Publicly available servers running web-based versions of these softwares are accessible at the following URL:<br>3D-JIGSAW: https://bmm.crick.ac.uk/~svc-bmm-3djigsaw/<br>SwarmDock: https://bmm.crick.ac.uk/~svc-bmm-swarmdock/index.html<br>Figures for all modelling data were generated in PyMOL v2.0.7 (Schrodinger LLC). Flow cytometry data was analyzed using FlowJo v10 (FlowJo LLC).<br>Epidermal microscopy was performed using Definiens Developer software (versionXD2.7)<br>Bioinformatics analysis was perfomed using Seurat (version 3.1.1.9023).<br>Graphs and statistical analysis were performed in Graphpad Prism 7 and Microsoft Excel. Figures were prepared using Microsoft Excel, Powerpoint and Adobe Illustrator.

For manuscripts utilizing custom algorithms or software that are central to the research but not yet described in published literature, software must be made available to editors/reviewers. We strongly encourage code deposition in a community repository (e.g. GitHub). See the Nature Research guidelines for submitting code & software for further information.

Data

Policy information about availability of data

All manuscripts must include a data availability statement. This statement should provide the following information, where applicable:

- Accession codes, unique identifiers, or web links for publicly available datasets
- A list of figures that have associated raw data
- A description of any restrictions on data availability

Fig 1c: raw flow cytometry plots are shown in Fig.1B
Fig 1f: example images are shown in Fig 1E
Fig. S1c: example images are shown in FigS1b
Fig. 2b: raw flow cytometry plots are shown in Fig2c
Fig 2h: raw flow cytometry plots are shown in Fig2g
Fig S3a: raw flow cytometry plots are shown in Fig 3a
Fig.4d : raw flow cytometry plots are shown in Suppl. Fig.3b
Fig.4e : raw flow cytometry plots are shown in Suppl. Fig.3c
Fig 5f: raw flow cytometry plots are shown in Fig 5c and Fig 5e
Supplementary Figure 5: Raw gene counts were obtained from GSE109413 (Moor et. al.) Ref 54 in manuscript and GSE92332 (Haber et. al.) Ref 55 in manuscript
https://www.mousephenotype.org/data/secondaryproject/3i

Data availability statement
This work did not include any data which mandated deposition in public databases. Associated raw data are provided in the main and/or supplementary figures. Relations to summary data charts are indicated and a full list of figures with associated raw data is provided in the reporting summary linked to this article. Raw gene counts were obtained from GSE109413 https://www.google.com/search?client=firefox-b-e&q=GSE109413 (Moor et. al.) 54 and GSE92332 https://www.ncbi.nlm.nih.gov/geo/query/acc.cgi?acc=GSE92332 (Haber et. al.) 55
For bioinformatics single cell analysis scripts are available on github: https://github.com/ajandke/Jandke_etal_naturecomms
Immunophenotyping data for pipeline procedure can be found under: https://www.mousephenotype.org/data/secondaryproject/3i

Field-specific reporting

Please select the one below that is the best fit for your research. If you are not sure, read the appropriate sections before making your selection.

☒ Life sciences ☐ Behavioural & social sciences ☐ Ecological, evolutionary & environmental sciences

For a reference copy of the document with all sections, see nature.com/documents/nr-reporting-summary-flat.pdf

Life sciences study design

All studies must disclose on these points even when the disclosure is negative.

Sample size	No statistical methods were used to predetermine sample size.<br>In vitro experiments were performed with replicates and repeated several times, based on previous experience with the protocols, and demonstrated high reproducibility. Sample sizes for individual experiments were dependent on genotypes of animals for different mouse strains and repeated indicated number of times. Due to comparison of multiple lines and in the interest of complying with the 3R n>=3 per genotype or group was not always available at analysis timepoints. Where data from multiple experiments are shown data are mean+/- SEM, where representative experiments are shown data are mean +-SD as indicated in the figure legends.
Data exclusions	No data were excluded
Replication	All experiments were successfully replicated independently (exact numbers indicated in legends). This included the use of different batches of cells and multiple cell lines, repeats performed across long periods of time, and similar experiments performed by multiple investigators.
Randomization	Experiments involved co-cultures and/or stainings of cell lines transduced with different defined constructs with or without additional treatments, and were taken from homogeneous pools. Allocation into experimental groups is irrelevant; Immortalized cells were harvested, counted and plated in equal numbers, prior to being tested with various experimental conditions, as described in the Methods and Figure legends.<br>For experiments involving cells isolated from primary mouse tissues, animals were picked randomly within the group described, adult (>4 weeks) males and females of different ages were used.
Blinding	Investigators performed, acquired and analysed experiments and as such were not blinded.Every sample was processed and analysed with the same criteria Blinding was not possible as experimental groups were determined by animals' genotypes.

Reporting for specific materials, systems and methods

We require information from authors about some types of materials, experimental systems and methods used in many studies. Here, indicate whether each material, system or method listed is relevant to your study. If you are not sure if a list item applies to your research, read the appropriate section before selecting a response.

Materials & experimental systems

n/a	Involved in the study
	x Antibodies
	x Eukaryotic cell lines
x	Palaeontology
	x Animals and other organisms
x	Human research participants
x	Clinical data

Methods

n/a	Involved in the study
x	ChIP-seq
	x Flow cytometry
x	MRI-based neuroimaging

Antibodies

Antibodies used

Name, clone name, manufacturer, catalogue number, lot number
CD3-APCCy7, 17A2, BioLegend,100222, B214134
TCRb-BV421 ,H57-597, BioLegend ,109229, B284306
CD122-PE,TM-b1, BioLegend, 123209, B263878
Thy1.2-BV510,53-2.1,BioLegend ,140319, B265837
Lag3-PerCPefluor710,C9B7W,eBioscience,46-2231-80, 2016887
CD24-BV650,M1/69,BD,563545, 8311910
CD8a-PECy7 53-6.7 BioLegend 100722, B255377
TCRVd4-FITC,GL-2,BD,552143, 7033856
TCRVd4-PE,GL-2,BioLegend,134905, B196042
CD8b PerCpCy5.5,YTS156.7.7,BioLegend,126610, B196260
TCRVg1.1/Cr4-FITC,2.11,BioLegend,141103,B268789
TCRVg4-APC,UC3-10A6, BioLegend, 137708, B192864
TCRd-BV421,GL3,BioLegend,118119, B290435
TCRd-Pe,GL3,BioLegend,118108, B178870
CD4-BV510,RM4-5,BioLegend,100559, B252816
TCR Vδ6.3/2-BV711, 8F4H7B7,BD744476, 9087604
CD25-PerCP/Cy5.5,PC61,BioLegend,102030, B198863
TCRVδ6.3/2 -PE,8F4H7B7,Pharmingen,555321, 37507
TCR Vg7,F2.67,Institut Pasteur, Paris, P.Pereira N/A
CD45Rb-FITC,C363.16A,eBioscience,11-0455-82, 1938748
Vg5-APC,7-17,BioLegend,137506, B261533
CD62L-BV421,MEL-14,BioLegend,104436, B238232
CD44-Pe-Cy7,IM-7,BioLegend,103030,B282420
CD45-eVolve605,30-F11, eBioscience, 83-0451-42, 1926764
TCR Vg5-PE, 536,BioLegend,137504, B191174
Vg5Vd1, 17D1 ,Yale, US, R.Tigelaar,J.Lewis N/A
TCR-Vg5-FITC,536, BD ,553229, 928598
MHC I-A/I-E-AF647, M5/114.15.2, BioLegend,107618, B216428
CD45-eFluor450, 30-F11, eBioscience ,48-0451-82, 4295770
Flag, M2 ,Merck, F1804, SLCC6485
Goat anti rat HRP, Thermo Fisher, 31470, JL1182407
Goat anti mouse HRP , Thermo Fisher, 31446, II10989614
CD69-Pe, H1.2F3, eBioscience, 12-0691-93, 1937997
CD45-PB, HI30, Biolegend, 304022, B263330
CD3-PercpCy5.5, SK7, Biolegend, 344808, B277411
TCRd-PerCPeFluor710, GL3, eBioscience, 46-5711-82, 201184
TCRd-AF647, GL3, Biolegend, 118134, B266832
DYKDDDDK-PeCy7, L5, Biolegend, 637324, B264498
HA-AF647, 16B12, Biolegend, 682404, B242905
HIS-PE, J095G46, Biolegend, 362603,362603

Flag-M2 magnetic beads, M8823, SLBV5095
Skint1(2G2) & Skint2 (3G8) Monoclonal Antibodies were generated at the core facility Helmholtz Zentrum Munich & tested on transfected cell lines at the FCI.

Validation	Commercial antibodies are validated by the manufacturer and can be traced by Lot numbers given above:<br>- Biolegend: https://www.biolegend.com/reproducibility<br>Biolegend declares that: Each lot of this antibody is quality control tested by immunofluorescent staining with flow cytometric analysis. This product lot has passed BioLegend's QC testing and is certified for use. For details on QC testing view our page at biolegend.com/en-us/quality-control. *BioLegend guarantees the performance of this product until the expiration date. The guarantee is dependent upon proper storage and handling as instructed on our Product Data Sheets. Every lot of product is quality tested against a "gold standard" reference lot. A new lot is only released based on our defined QC specifications to ensure lot to lot reproducibility and reliability. BioLegend guarantees the stability and performance of all our products shipped at room temperature."<br>- ThermoFisher (including eBioscience): https://www.thermofisher.com/uk/en/home/life-science/antibodies/invitrogen-antibody-validation.html? icid=ab-search-learning-ab-validation<br>- R&D Systems: https://www.rndsystems.com/products/rd-systems-approach-antibody-quality<br>-BD: The details of the process on how we determine antibody specificity are company proprietary (according to Vesna Melkebeek, European Scientific and Technical Support Specialist)<br>Non-commercial antibodies were validated in the references provided:<br>-Vg7: Di Marco Barros, R. et al. Epithelia Use Butyrophilin-like Molecules to Shape Organ- Specific gammadelta T Cell Compartments. Cell 167, 203-218 e217 (2016).

Eukaryotic cell lines

Policy information about cell lines

Cell line source(s)	Francis Crick Institute (FCI) Cell Services:<br>293T: originated from the HPA Cultures Cell Bank,<br>J76: Jurkat E6.1 subline, TCRalpha(neg) TCRbeta(neg),<br>MODE-K: gift from Dr. D. Kaiserlian, INSERM U1111, Lyon, France
Authentication	Authentication performed by the ATCC and FCI Cell Services by STR profiling and species identification. J76 lines were TCRab (neg) and CD3(neg). CD3 cell surface expression was restored following transduction with TCRgd constructs.
Mycoplasma contamination	Cell lines were confirmed negative for mycoplasma by FCI cell services
Commonly misidentified lines<br>(See ICLAC register)	No commonly misidentified cell lines were used in this study

Animals and other organisms

Policy information about studies involving animals; ARRIVE guidelines recommended for reporting animal research

Laboratory animals	Mus musculus.<br>Strains commercially available are: Btnl1(KOMP) UCDavis, KOMP repository project ID: CSD 67994, Btnl4(KOMP) UCDavis, KOMP repository: project ID: CSD81524. The phenotyping of the Btnl1-KOMP and Btnl4-KOMP mice has previously been published: Di Marco Barros, R. et al. Epithelia Use Butyrophilin-like Molecules to Shape Organ-Specific gammadelta T Cell Compartments. Cell 167, 203-218 e217, doi:10.1016/j.cell.2016.08.030 (2016).<br>The Skint1Tac line was published: Lewis, J. M. et al. Selection of the cutaneous intraepithelial gammadelta+ T cell repertoire by a thymic stromal determinant. Nat Immunol 7, 843-850, doi:ni1363 [pii]. 10.1038/ni1363 (2006).<br>The Skint1-KO line was published: Narita, T., Nitta, T., Nitta, S., Okamura, T. & Takayanagi, H. Mice lacking all of the Skint family genes. Int Immunol, doi:10.1093/intimm/dxy030 (2018).<br><br>FVB & NF-Skint1-Tg mice were previously described: Barbee, S. D. et al. Skint-1 is a highly specific, unique selecting component for epidermal T cells. Proc Natl Acad Sci U S A 108, 3330-3335, doi:1010890108 [pii] 10.1073/pnas.1010890108 (2011).<br>Unless stated otherwise, adult (age: 6+ weeks) male & female mice were used for all strains in this study.<br><br>Btnl deleted (Btnl1-DD, Btnl1Dgut, Btnl6DD, Btnl6Dgut, Btnl146Indel) mouse strains described in this paper were generated at the FCI in accordance with local guidelines and maintained in under described conditions.<br><br>Unless otherwise stated adult mice (age 4+weeks), male and female of all strains of mice were used in this study.
Wild animals	No wild animals were used
Field-collected samples	No field-collected samples were used
Ethics oversight	All animal work was carried out under project license: 7009056 to A.C.H. and individual experimental protocols were approved by the FCI NACWO.

Note that full information on the approval of the study protocol must also be provided in the manuscript.

Flow Cytometry

Plots

Confirm that:

- [x] The axis labels state the marker and fluorochrome used (e.g. CD4-FITC).
- [x] The axis scales are clearly visible. Include numbers along axes only for bottom left plot of group (a 'group' is an analysis of identical markers).
- [x] All plots are contour plots with outliers or pseudocolor plots.
- [x] A numerical value for number of cells or percentage (with statistics) is provided.

Methodology

Sample preparation	Mouse IEL were isolated from small intestine as previously described (Di Marco Barros, Cell, 2016). Briefly, small intestine was opened, washed in PBS, cut into 0.5 cm long pieces and incubated at RT on a wheel in complete RPMI supplemented with 1mMDTT. Tissues were then washed, vortexed in complete RPMI and filtered through 70 nm nylon cell strainers. Vortexing and filtration steps were repeated twice. IEL were then purified by Percoll density centrifugation.
Instrument	Fortessa flow cytometers (BD)
Software	DIVA (Acquisition) and FlowJo v10 (analysis).
Cell population abundance	Cells were not sorted for this study.
Gating strategy	For primary IEL FSC-A, SSC-A (lymphocytes)/ FSC-H, FSC-H SSC-H/SSC-W (singlets)/Live-dead negative (viable cells)/ CD3+ TCRb-/ Vg7+ or Vg7- cells were gated.<br>For primary IEL co-culture: FSC-A, SSC-A (lymphocytes)/ FSC-A, FSC-H (singlets)/Live-dead negative (viable cells)/ CD3+ TCRb-/ Vg7+/ Vd4+ or Vd6.3+/CD25+.<br>For J76 co-culture: FSC-A, SSC-A (lymphocytes)/ FSC-A, FSC-H (singlets)/ GFP- (to exclude 293T cells)/CD45+/TCRgd+ Vg7+/CD69 +.<br>Representative raw primary flow plots are provided for most experiments.<br>Gating plots are provided in Supplementary Figure 6.

- [x] Tick this box to confirm that a figure exemplifying the gating strategy is provided in the Supplementary Information.
